# Injuries in Netball-A Systematic Review

**DOI:** 10.1186/s40798-020-00290-7

**Published:** 2021-01-06

**Authors:** Christopher Downs, Suzanne J. Snodgrass, Ishanka Weerasekara, Sarah R. Valkenborghs, Robin Callister

**Affiliations:** 1 School of Health Sciences, University of Newcastle, Newcastle, NSW Australia; 2 Priority Research Centre for Physical Activity and Nutrition, University of Newcastle, Newcastle, NSW Australia; 3 Department of Physiotherapy, University of Peradeniya, Peradeniya, Central Province Sri Lanka; 4 University of Newcastle, School of Biomedical Science and Pharmacy, Newcastle, NSW Australia

**Keywords:** Netball, Injuries, Systematic review, Epidemiology, Sport, Ankle injuries, Knee injuries

## Abstract

**Background:**

Netball is estimated to be played by more than 20 million people worldwide, but there is evidence of high injury incidence. A thorough understanding of the types and rates of netball injuries is essential for effective injury management and prevention strategies to be developed and implemented. This systematic review summarises the published findings with respect to injury types, participant characteristics and any identified risk factors for netball injuries.

**Methods:**

A librarian-assisted computer search of seven scientific databases was conducted for studies reporting on netball injuries. Inclusion criteria were studies published in English, in peer-reviewed journals, which reported data on injuries and variables (e.g. age and competition level) that have been proposed as possibly associated with netball injury risk.

**Results:**

Forty-six studies (43.5% prospective, 37% hospital/insurance records, 19.5% retrospective) from 45 articles were included after screening. The majority of studies (74%) were conducted in Australia or New Zealand. There was little consistency in the definition of ‘injury’. Elite or sub-elite level players were included in 69% of studies where the level of competition was reported. The duration of injury surveillance was generally related to the format of competition from which data were collected. Self-report questionnaires were used in 48% of studies and only 26% of studies used qualified health professionals to collect data courtside. Injuries to the ankle and knee were the most common (in 19 studies) although the incidence varied considerably across the studies (ankle 13–84% and knee 8–50% of injuries). Prevention of ankle and knee injuries should be a priority. Children sustained more upper limb injuries (e.g. fractures) compared with adults who sustained more lower limb injuries (e.g. ankle and knee sprains/strains). A large number of potential risk factors for injury in netball have been investigated in small numbers of studies. The main circumstances of injury are landings, collisions and falls.

**Conclusion:**

Further studies should be directed towards recreational netball, reporting on injury incidence in players by age and utilising high-quality, standardised methods and criteria. Specific injury diagnosis and a better understanding of the circumstances and mechanisms of injury would provide more meaningful data for developing prevention strategies.

**Supplementary Information:**

The online version contains supplementary material available at 10.1186/s40798-020-00290-7.

## Key Points

- Injury rates increase with increasing age, and the most common sites of injury change from the upper limb (including fractures to the arm, wrist and hand) in those < 16 years to the lower limb (particularly ankle and knee sprains and strains) in adults.
- Existing evidence suggests that injury rates appear higher in elite players (19.35/1000 PH) than recreational players (11.3–14/1000 PH) and in matches than in training.
- Common circumstances of injury are jump-landing, trips/slips/falls and contact with another player or the ball.
- More centralised national databases incorporating quality injury diagnosis across all competition levels would improve our understanding of injuries in netball.

## Introduction

The International Netball Federation (INF) has estimated that more than 20 million people play netball in more than 70 countries [1]. Netball is played predominantly in British Commonwealth countries and usually by females, although there is growing participation by males in both male and mixed competitions [2]. Players typically range in age from 5 to over 50 years and games are played on both indoor and outdoor courts, with a variety of court surfaces [3].

While netball is a sport with one of the highest participation rates, there is also evidence of high injury rates, with comparisons being made to other team sports including all football codes, basketball, field hockey and cricket [4–9]. Injuries are a concern in netball and injury prevention is a goal of the sport’s governing bodies (INF; Netball Australia [10]). Injury surveillance is the first stage of the Translating Research into Injury Prevention Practice framework (TRIPP) [11]. Surprisingly there has been no published review synthesising the available data on netball injuries alone. A thorough understanding of the types and rates of netball injuries is essential in order to develop appropriate injury prevention and management strategies. Therefore, the purpose of this systematic review is to identify, collect and synthesise all the published data on netball injuries with respect to injury types, participant characteristics and any identified factors associated with the risk of injuries in netball.

## Methods

The conduct and reporting of this review adheres to PRISMA guidelines [12].

### Study Eligibility Criteria

Studies were included if they reported data on netball injuries sustained by netball players of any age, sex or competition level, were full-text peer-reviewed articles and published in English. Included study designs were observational and experimental. Case studies, reviews and conference abstracts were excluded. Included studies reported data on netball injuries in general and/or in relation to variables such as age, competition level or any other factors associated with netball injury or injury risk. Studies were included regardless of injury definition; i.e. any injury definition was acceptable provided the injury was related to netball matches or training.

### Search Strategy

A biomedical librarian assisted with the development of the search strategy for a systematic search of the electronic databases Cinahl, Medline, Embase, Cochrane, Informit Health, Scopus and SPORTDiscus from 1985 to 20th January 2020 (completed on 20th January 2020). The search was limited to 1985 as the paper by Hopper [13] is regarded as the first peer-reviewed publication of a study of netball injuries. In an effort to be as comprehensive as possible, the search strategy used only the terms Netball* AND Injur*. A secondary search of the reference lists of included papers was conducted to identify additional articles for possible inclusion.

### Study Selection and Quality Assessment

After removal of duplicates, two reviewers independently screened titles and abstracts. If there was uncertainty regarding inclusion of identified articles, the full text was obtained and screened. Discrepancies were first independently re-assessed by each reviewer, then resolved following independent assessment by a third reviewer. Agreement between reviewers was calculated using Cohen’s Kappa [14]. The quality of the studies was assessed using the NIH Quality Assessment Tool for Observational Cohort and Cross-Sectional Studies [15].

### Data Extraction and Synthesis

Data from the included studies were extracted by one reviewer and verified by a second in a database template. Data included quality assessment information, country of origin, participant characteristics (age, competition level), data collection period and methods, injury definition, player exposure, injury rate or proportion, injury site, type and severity and key study findings. Results of studies were summarised qualitatively for comparison. Quantitative assessment was limited to tallying the number of studies reporting a particular issue.

## Results

### Study Selection

**Fig. 1:**
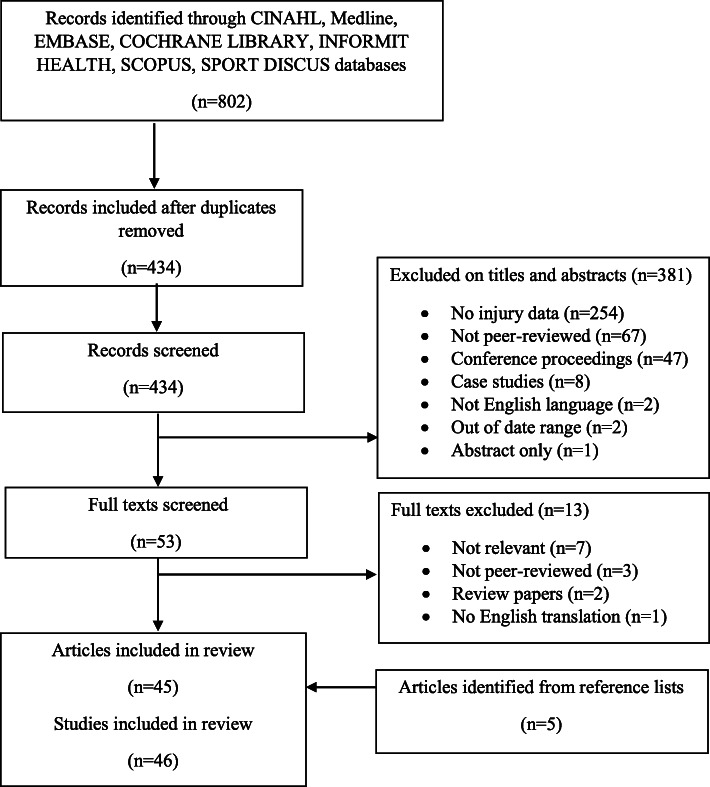
Search results and selection of studies

The search identified 434 articles after duplicates were removed. After screening, 45 articles were included in the review (Fig. 1). The inter-reviewer agreement for the title/abstract and full-text screenings was considered to be good (*κ* = 0.59, 95% CI: 0.51–0.68) and very good (*κ* = 0.87, 95% CI: 0.74-1.01), respectively. The main reasons for exclusion were that no injury data were reported or publication was not peer-reviewed. Only two articles [13, 16] were published between 1985 and 1989, with 11 in the 1990s, 14 in the 2000s, and 18 in the 2010s. One article [17] reported on two studies (one prospective, one retrospective); therefore, there were 46 studies. Langeveld et al. [18] and Coetzee et al. [19] used data from the same multiday tournament study. Hopper and Elliot [17] and Hopper [20] also used the same data source as one another but they reported on different research aims. There was overlap in the data used in Stevenson et al. [21] (one season) and Finch et al. [22] (two seasons). Also, there was an overlap in the data used in the articles of Hume [23] and Hume and Marshall [24].

### Publication Quality

**Fig. 2:**
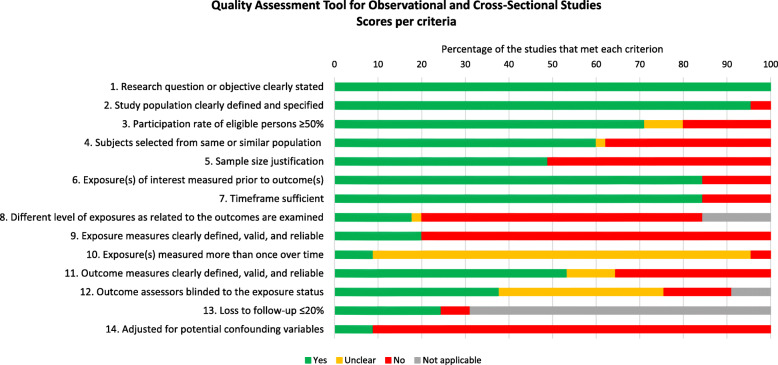
Quality assessment of included papers

All studies included in this review had a clearly stated research question or objective (Fig. 2) and most of the studies (96%) clearly defined their study population. The participation rate of eligible players was adequate (i.e. > 50%) in 71% of studies. The time frame of exposure to netball was reported in 84% of studies. Sample size justification was reported in only 49% of studies. Exposure levels were clearly defined and determined using valid and reliable methods in 20%, but only reported in 18% of studies. Statistical adjustment (e.g. regression) for confounding variables (e.g. playing and training time) was adequate in only 9% of studies. The quality assessment results per individual study and criterion are detailed in Supplemental Table 1.

### Review Findings

Table 1 provides a summary of country of origin, sample size, participant age, competition level, and the type, duration and methods of data collection in the included studies.

**Table 1:** Characteristics of included studies (*n* = 46) documenting injuries to netball players

Study	Country of origin	Sample	Age (years) Mean ± SD or range	Competition level	Data collection period	Data collection methods	Injury data collected
**Prospective season competition**							
Attenborough et al. 2017 [25]	AUS	94 participants 11 ankle injuries	21.5 ± 6.3	Elite/Inter-district/recreational (club)	1 × season for a player	Elite/Inter-district reports (injury/exposure) by team physio/club director; Recreational self-report; Follow-up by phone; Preseason data collection in one session.	Ankle only
Elphinston et al. 2006 [26]	UK	17 participants 22 vs 4 injuries	25.9 ± 2.6	Elite	~ 2 × seasons	Physiotherapy assessment.	All injuries
Ferreira et al. 2010 [27]	SA	25 participants 46 injuries	18-23	Elite	1 × season	Weekly physiotherapy clinic assessment.	All injuries
Finch et al. 2002 [22]	AUS	247 participants 216 netball injuries	~ 22 ± 8	Recreational (club)	2 × 5-month seasons per player	Baseline questionnaire; diary record then self-report by monthly telephone interview over each season.	All injuries
Hopper 1986 [13]	AUS	3108 participants 158 netball injuries	12–15 > 15	Competitive to Recreational (club)	1 × 14-week season 1983	Injury data recorded in questionnaire by courtside first aid room physiotherapist; other data provided by player in self-report questionnaire.	All moderate to severe injuries
Hopper et al. 1995 [28]	AUS	11228 participants 608 netball injuries	> 14 18.8 ± 5.6	Competitive to Recreational (club)	5 × 14-week seasons 1984–1989	Injury data recorded in questionnaire by courtside first aid room physiotherapist; other data provided by player in self-report questionnaire.	All moderate to severe injuries
Hopper et al. 1995 [29]	AUS	72 participants 22 netball injuries	15–36 20.6 ±3.6	Elite to Recreational (club)	1 × 14-week season 1989	Preseason lab testing: somatotype, hypermobility, vertical jump, anaerobic fitness (10-s cycle). Injury data recorded in questionnaire by courtside first aid room physiotherapist; other data provided by player in self-report questionnaire; training injuries by player report to physiotherapist.	Lower limb and back only
Maulder et al. 2013 [30]	SA	24 participants 9 netball injuries	21.6 ± 3.2	Elite (national) Sub-elite (regional)	1 × 6-month season	Player self-report fortnightly via email/phone questioning Lower limb dominance and asymmetry assessments during preseason.	Lower limb only
McKay et al. 1996 [31]	AUS	9,190 participants 159 netball injuries	27.2 ± 7.8	Elite/Recreational (club)	2 × seasons 1991–1992	Courtside injury observers (physiotherapy students/St John’s Ambulance First Aiders) Questionnaire completed by players; Phone follow-up to obtain treatment sought and time loss.	All injuries
McManus et al. 2006 [32]	AUS	368 participants 272 netball injuries	Majority (66%) 16–30	Recreational (club)	2 × seasons 1997–1998	Baseline questionnaire then telephone interviews every 4 weeks throughout season.	All injuries
Pickering Rodriguez et al. 2017 [33]	AUS/NZ	29 participants 12 lower body netball injuries	24.1 ± 3.2	Elite/Sub-elite	1 × season 2013	Injuries: Elite—team physiotherapist; Sub-elite—self-report. Preseason lower limb stiffness measures.	Lower body non-contact injuries
Pringle et al. 1998 [34]	NZ	1512 participants 15 netball injuries	6–15	Recreational (club)	4 weeks of season	Trained observers (physiotherapy or sports science students) observed games and recorded injuries on incident form over 4 weeks; Post-game interview with player; Telephone follow-up by physiotherapist within 3 days and then after one week (diagnosis and time loss).	All injuries
Stevenson et al. 2000 [21]	AUS	258 participants 112 netball injuries	22 ± 8 (13.6-30.4)	Recreational (club)	1 × 5-month season 1997	Baseline questionnaire then telephone interviews every 4 weeks throughout season.	All injuries
Zulkarnain et al. 2019 [35]	Malaysia	42 participants Intervention (IG) *n* = 17 Control (CG) *n* = 25 31 injuries	IG 21. ± 2 1.8 CG 20.9 ± 1.9	Club/District 23.9% State 60.9% National 15.2%	6 weeks post intervention	Study compared 6-week safe-landing technique injury prevention program (Down 2 Earth) to standard training program; injuries recorded for 6 weeks following the intervention period.	Lower limb injuries
**Prospective tournaments**							
Hopper and Elliot 1993 [17]	AUS	228 participants 52 netball injuries	U16 14.8 ± 0.4 U21 19.2 ± 2.2 Open 23.7±3.6	Elite/Sub-elite	Multi-day Tournament 1988	Player baseline questionnaire and podiatric assessment of lower limbs and back. Injuries assessed and recorded by physiotherapists; players completed questionnaire regarding events relating to the injury.	Lower limb and back injuries only
Hopper 1997 [20]	AUS	213 participants 52 netball injuries	U16 14.8 ± 0.4 U21 19.2 ± 2.2 Open 23.7±3.6	Elite/Sub-elite	Multi-day Tournament 1988	Player baseline questionnaire and podiatric assessment of lower limbs and back. Injuries assessed and recorded by physiotherapists; players completed questionnaire regarding events relating to the injury; somatotype assessed.	Lower limb and back injuries only
Hume et al. 2000 [36]	AUS	940 participants 131 netball injuries	18.4 ± 4.4 U17, U19, U23, Open	Sub-elite	Multi-day Tournament (State)	Injured players completed a 5-page questionnaire; Injury treatment provided by physiotherapists, sports trainers, St John’s First Aid personnel, Sports Medicine Australia Van personnel, coaches, or self-treatment.	All injuries
Langeveld et al. 2012 [18]	SA	1280 participants 205 netball injuries to 192 players	U19, U21, Open, USSA	Elite/Sub-elite	3 × Multi-day Tournaments 2009	Injury questionnaire completed by specifically-trained medical staff, team managers or coaches.	All injuries
Coetzee et al. 2014 [19]	SA	1280 participants 205 netball injuries to 192 players	U19, U21, Open, USSA	Elite/Sub-elite	3 × Multi-day Tournaments 2009	Injury questionnaire completed by specifically-trained medical staff, team managers or coaches and questionnaire completed by players re training history and injury prevention practices.	All injuries
Smyth et al. 2019 [37]	AUS	192 participants 103 injuries to 80 players	U17 U19	Pre-elite	6 Day Tournament 2018	Medical attention injuries diagnosed by physiotherapists and recorded whether the athlete could continue to play immediately after the injury. Players completed a Health Problems Questionnaire which provided additional indications of injuries and effects.	All injuries
**Retrospective**							
Attenborough et al. 2016 [38]	AUS	96 participants 69 previous ankle injuries	ID 19.4 ± 3.5 Club 24.1 ± 7.9	Inter-district/Recreational (club)	All previous ankle injuries	Participant questionnaire including ankle sprain history; assessment with Cumberland Ankle Instability Tool—Youth and ankle joint laxity (arthrometer).	Recurrent ankle injuries
Finch et al. 2006 [39]	AUS	1084 residents 648 all sports/activities 34 netball injuries	> 5	Recreational	All sport or active recreation injuries in previous 2 weeks	Household telephone survey.	All injuries
Hopper and Elliot 1993 [17]	AUS	228 participants 504 netball injuries	U16 14.8 ± 0.4 U21 19.2 ± 2.2 Open 23.7±3.6	Elite/Sub-elite	All previous lower limb and back injuries	Questionnaire: injury history, age, playing position, playing experience, use of orthosis, perceived reasons for injury Podiatric assessments of lower limb and back, including foot type.	Lower limb and back injuries
Hopper et al. 1994 [40]	AUS	204 participants 188 injured players 449 lower limb injuries	U16 14.8 ± NR U21 19.1 ± NR Open 23.7 ± NR	Elite/Sub-elite	All previous lower limb injuries	Questionnaire and foot assessments as per Hopper et al. 1993. 5 categories of foot types: normal, pronating with rearfoot abnormalities, pronating with forefoot abnormalities, non-pronating, other.	Lower limb injuries
Pillay et al. 2012 [41]	SA	254 participants 301 netball injuries 157 players injured	Nat 24.3 ± 6.3 State 23.9± 5.1 Club 24.2 ± 4.3	Elite/Sub elite	All injuries in previous season	Questionnaire completed by netball players at tournament.	All injuries
Singh et al. 2013 [42]	Jamaica	59 participants 70 netball injuries	U16, U21, Senior	Elite	All previous injuries	Questionnaire—self-reported player characteristics, injuries, factors associated with injury.	All injuries
Smith et al. 2005 [43]	AUS	200 participants 69 players injured	6–16 11 ± 2.5	Competitive to recreational (club)	All previous injuries	Questionnaire—self-reported player characteristics, injuries, preventative equipment use Joint hypermobility assessed using Beighton index.	All injuries
Stuelcken et al. 2016 [44]	AUS	16 elite players with ACL injuries	Not reported	Elite	Televised games	Medically diagnosed injury; Video analysis of circumstances of injury.	ACL injuries
Whatman et al. 2017 [45]	NZ	166 female participants	16 ± 1	Secondary school	Overuse knee and ankle injuries in previous 12 months	Oslo Sports Trauma Research Centre Overuse Injury Questionnaire re knee (*n* = 106 completed) and ankle (*n* = 113 completed).	Knee and ankle overuse injuries
**Hospital, clinic or insurance records**							
Cassell et al. 2003 [46]	AUS	2300 all sports 81 netball to ED 67 netball to GP	> 4	Mostly recreational	1 year	Netball-related ED presentations from Victorian Injury Surveillance System. Netball-related GP presentations from Extended Latrobe Valley Injury Surveillance (ELVIS) project.	ED and GP presentations
Chong et al. 2004 [47]	Singapore	13 female ACL injuries 4 netball injuries	13–38	School/Club	4 years	Female ACL injuries at regional Singapore hospital	Female ACL injuries
Fernando et al. 2018 [9]	AUS	5483 netball injury presentation to ED	> 5	All	3 years Mid 2012–Mid 2015	Presentations of Victorian Emergency Minimum Dataset in Victoria NSW.	All presentations
Finch et al. 1998 [6]	AUS	2,165 child netball injuries 3,098 adult netball injuries	< 15 > 15	Mostly recreational	5 years	Sport and active recreation injury cases at selected hospital emergency departments from 1989–1993; standardised data collection form completed by injured player (age, sex, sport, context of injury) and treating doctor (diagnosis, treatment details).	Emergency department injury cases
Flood et al. 2009 [48]	AUS	4,596 netball injuries	> 5 26.3 ± 10.9	All	5 years 2000–2004	Netball-related hospital admissions from the National Hospital Morbidity Database; includes data from all public and most private hospitals.	Hospital admissions
Gwynne-Jones et al. 2011 [49]	NZ	363 Achilles injuries 285 sports injuries 88 netball injuries	15–60	All	8.5 years 1999–2008	Participants identified through emergency department, in patient, surgical audit and physiotherapy department records.	Achilles tendon rupture
Hassan et al. 2001 [50]	UK	54 netball injuries 13 netball fractures	5–15	Children	1 year 1997–1998	District General Hospital accident and emergency department.	Fractures
Hon et al. 2001 [51]	Malaysia	113 sports injuries 3 netball injuries	7–59	Competitive/ recreational	1 year 1998–1999	Patients reporting to Department of Orthopaedic and Traumatology of Seremban Hospital with fractures sustained during sporting activity.	Fractures
Hume 1993 [23]	NZ	143 hospitalisations 284 netball in ED 1420 insurance claims 298 sports injury clinic	5–15 > 15	All	Most 1 year Clinic 1.7 years	Multiple sources: hospitalisation morbidity data (1 year); Dunedin Hospital Emergency (A&E) Department data (1 year); Accident Compensation Corporation (insurance) claims (1 year); Dunedin Sports Injury Clinic data (1.7 years). Population and netball participation data from government sources.	All presentations and claims
Hume et al. 1994 [24]	NZ	139 hospitalisations No fatal injuries 3.2% of all injuries 89.3 injuries/100,000 1420 Insurance claims—netball 5.3% 279 ED—netball 7.6% 169 Sports clinic—netball 27.3%	All	All	Mostly 1 year Mortality 10 years	Multiple sources: HIS mortality data (10 years); hospitalisation morbidity data (1 year); Dunedin Hospital Emergency (A&E) Department data (1 year); Accident Compensation Corporation (insurance) claims (1 year); Dunedin Sports Injury Clinic data (1 year).	All presentations and claims
Joseph et al. 2019 [3]	AUS	1215 approved insurance claims	34 ± 17	All	1 year 2016	Netball Australia National Risk Protection Accident Insurance Program data for 2016. Covers all registered players in competitions organised by Netball Australia. Covers training and matches.	All approved claims
King et al. 2019 [52]	NZ	11748 mod-serious Females 10,061 Males 1687 9 serious claims (all female)		All	5 years 2012–2016	NZ Accident Compensation Corporation data over 5 years on acute personal injury claims. Data collected in standardised manner when present to medical practitioner.	Moderate to serious and serious injuries
Kirkwood et al. 2019 [8]	UK	*N* = 154 netball attendances (98% female)	0–19	All	2.25 years Jan 2012–Mar 2014	Injuries incurred during netball participation.	All ED attendances
Love et al. 1998 [53]	NZ	260 netball dental insurance claims	0–75+	All	4 years 1993–1996	New Zealand Accident Rehabilitation and Compensation Insurance Corporation (ACC) database of dental claims related to sports injuries for 4 years.	Dental claims
Otago et al. 2007 [54]	AUS	829 netball injuries	> 10	All	1 year 1999	Insurance claims (accepted) for netball injuries over 12 months.	Insurance claims
Purdam 1987 [16]	AUS	20 Netball participants 105 netball injuries	Not reported	Elite	0.83 year 1986	Physiotherapy department records at the Australian Institute of Sport	All treated
Smartt et al. 2009 [55]	NZ	1126 netball injuries Male 215; Female 911	5–82 29 ± NR	All	6 years 2000–2005	Hospital inpatients related to netball injury; data also linked to injury entitlement claims Participation in netball estimated from national survey data.	Hospital admissions

*ACL* anterior cruciate ligament; *AUS* Australia; *ED* emergency department; *GP* general (primary) practitioner; *NZ* New Zealand; *SA* South Africa; *U* under (age); *UK* United Kingdom; *USSA* University Sports South Africa

#### Country of Origin of Study

Of the 46 studies, all were conducted in British Commonwealth countries with 26 (57%) in Australia, 8 (17%) in New Zealand, 5 (11%) in South Africa, 3 (6.5%) in the UK, 2 (4.3%) in Malaysia and 1 (2.2%) each in Singapore and Jamaica.

#### Study Categories

The studies are presented in Table 1 in four categories: prospective studies over one or more playing seasons (*n* = 14, 30%), prospective studies at tournaments (*n* = 6, 13.0%), retrospective studies (*n* = 9, 20%) and studies accessing database sources, including hospital and insurance company records (*n* = 17, 37%). One article [17] reported both prospective and retrospective studies and the findings from these data are reported in their respective sections.

#### Number of Participants and Injuries

Among prospective studies during competition seasons, participant numbers ranged from 17 to 11,228, with 50% (*n* = 7) of studies having< 100 participants. Injury numbers ranged from 11 to 608, with 43% (*n* = 6) reporting < 25 injuries. In prospective studies over the course of a tournament, participant numbers ranged from 192 to 1280, and injury numbers from 52 to 205. In the retrospective studies, participant numbers ranged from 16 to 1084; 88% (*n* = 7) of studies had < 254 participants. Injury numbers ranged from 16 to 504 with most studies reporting < 100 injuries. Injury numbers in the hospital, clinic and insurance records studies ranged from 3 to 5,263 with nine of the studies reporting > 1000 injuries.

#### Age of Participants

Age range or mean age were reported in most studies. Among the prospective studies, only Pringle et al. [34] focused on children < 16 years, whereas many studies [13, 17, 20, 28, 29, 36] included participants > 12 years old, with the mean age typically in the 20–25-year range. In the retrospective studies, Smith et al. [43] included only those 6–16 years and Whatman and Reid [45] investigated secondary school participants (16 ± 1 years) whereas the other studies covered wider age ranges [45]. Hassan et al. [50] in their hospital ED admission study focused on children 5–15 years whereas the other large database reports included both children and adults. However, these studies limited their reporting of results related to age [3, 9, 17, 42, 48, 54, 55] to only ‘child versus adult’ limiting the extraction of injury by specific age brackets. Although some studies did not report age specifically, it was inferred from the level of competition, which was often played in age categories in those studies.

#### Sex of Participants

Seven studies indicated that they included males, who represented 5.7–10.5% of hospitalisations [21–23], and 2–19% of injuries [52] [48, 52, 55]. No other information was provided on the sex of participants, although it was presumed to be only female in most studies, based on the level of competition or competition categories.

#### Level of Competition

Participants in the included studies were elite in five (11%) studies [16, 26, 27, 42, 44], elite and/or sub-elite in another 11 (24%) studies [17–20, 30, 33, 36, 37, 40, 41]. In 13 (28%) of the studies [6, 13, 21, 22, 28, 32, 34, 39, 45–47, 50, 51] participants were not elite, and in the final 17 (37%) studies [3, 8, 9, 23–25, 29, 31, 35, 38, 43, 48, 49, 52–55] participants were from a wide range of competition levels.

#### Data Collection Period

Of the prospective studies, six (30%) were conducted over less than one week (tournaments) [17–20, 36, 37]. Two (10%) of these studies were over part of a season [34, 35], seven (35%) were over one season [13, 21, 25, 27, 29, 30, 33], and five (25%) were over two or more seasons [22, 26, 28, 31, 32]. The retrospective studies (*n* = 9) investigated injuries in the previous two weeks [39], previous season [41], previous year [45], during televised games [44] or all previous injuries [17, 38, 40, 42] [43]. The hospital/clinic/insurance records analyses were conducted over 10 months (*n* = 1) [16], 1 year (*n* = 6) [3, 23, 46, 50, 51, 54], 2–3 years (*n* = 2) [8, 9] or 4 or more years (*n* = 8) [6, 24, 47–49, 52, 53, 55].

#### Data Collection Methods

In the prospective studies, physiotherapists diagnosed and recorded injuries in 10 (50%) studies [13, 17, 20, 25–29, 33, 37], although in three of these studies, only elite players’ data were provided by physiotherapists, with non-elite players’ data provided by self-report [25, 33, 37]. Five (25%) of the prospective studies [21, 22, 30, 32] used only self-report data, and training injuries were often dependent on player notification to a therapist [29]. In the other prospective studies, physiotherapists or students/trainers/first aiders/coaches provided injury treatment and records of injury [18, 19, 31, 34, 36]. In the retrospective studies, eight (89%) studies [17, 38–43, 45] relied exclusively on self-report of injuries while one study [44] used medical diagnosis of anterior cruciate ligament (ACL) injuries. Health professionals provided the diagnoses and injury records for all the hospital/clinic/insurance records studies.

#### Sites and Severity of Injury Data Collected

In the prospective studies, 11 (55%) studies [18, 19, 21, 22, 26, 27, 31, 32, 34, 36, 37] investigated all injuries. Two (10%) studies investigated all moderate-severe injuries (injury required immediate treatment and resulted in some disability) [13, 28]. Three (15%) studies focused on lower limb and back injuries [17, 20, 29], two lower limb only [30, 35], one lower body non-contact injuries [33] and one only ankle injuries [25]. Of the retrospective studies, four (44%) investigated all injuries [39, 41–43]. One study investigated recurrent ankle injuries [38], one lower limb and back [17], one lower limb [40], one knee and ankle overuse [45] and one ACL injuries [44]. In the hospital/clinic/insurance records studies, 12 (71%) investigated all injuries [3, 6, 8, 9, 16, 23, 24, 46, 48, 52, 54, 55], two (12%) focused on fractures [50, 51], one on ACL injuries [47], one on Achilles ruptures [49] and one on dental claims [53].

### Results Regarding Injury Characteristics

Table 2 provides a summary of the injury definitions used, player exposure and injury rates, the primary sites and types of injuries, and the key findings of the studies.

**Table 2:** Characteristics of injuries to netball players reported in included studies (*n* = 46)

Study	Injury definition	Exposure^**a**^	Injury rate or proportion of injuries	Injury site	Type and/or severity of injury	Key findings^b^
**Prospective season competition**						
Attenborough et al. 2017 [25]	Ankle injury time loss >1d	Matches and training Matches 1333 h Training 4992 h Total 6325 h	6.75 sprains/1000 PH matches 0.40 sprains/1000 PH training 1.74 sprains/1000 PH total	Ankle	Ankle sprains	11 sprains: 9 during matches; 2 during training Odds ratio for ankle sprain 4.04 × higher (*p* = 0.04) if reach distance in post-medial direction of SEBT < 77.5% of leg length; trends for relationships between ankle joint laxity, static and dynamic balance and injury risk; no relationships with previous sprain, vertical jump performances or perceived ankle instability
Elphinston et al. 2006 [26]	Not stated	Not reported	1.3 injuries/player at baseline 0.2 injuries/player after intervention	Pre v. Post intervention: Ankle—27 vs 25% Knee—14 vs 50% Lower back —18 vs 0% Shoulder—32 vs 25% Neck—9 vs 0%	Any	Implementation of a multidisciplinary integrated sports science and sports medicine approach addressing fundamental functional problems of players resulted in a marked reduction in the number of injuries. Screening and evaluation of players functional performance followed by individualised technical training and conditioning were implemented resulting in a marked reduction (550%) in injuries. No training or overuse injuries after intervention.
Ferreira et al. 2010 [27]	Injury sustained during match or training	Not reported	1.84 injuries/player	Ankle—39.1% Knee—28.3% Cervical—8,7%	Minor—35% Moderate—56.6% Severe—8.7%	35% of injuries minor; 56.5% moderate (miss 8–21 d); 8.7% severe. 52.2% of injuries associated with incorrect landing. Inadequate physical or motor abilities (biomechanical deviations, excess body fat, poor balance, and limited explosive power) were identified and may have contributed to injuries.
Finch et al. 2002 [22]	Injury led to reduced sporting activity or treatment or adverse economic/social effects	68% training and matches< 2.5 h per week	11.3 (95%CI 9.8–12.9)/1000 PH total	Ankle—53.8% Knee—27.7% Finger/thumb 26.2% Lower back—16.9% Head/face 6.2%	Muscle strain—38.8% Contusion—24.8% Ligament sprain—61.2% Broken bone—8.5% Moderate—60–70%	Ligament sprains/tears and ankle injuries more common in netball than many other sports. Most injuries (60–70%) were classified as moderate severity and required treatment by a health professional. Injury rate in netball lower than AFL, field hockey and basketball. 94.3% of netball players were female.
Hopper 1986 [13]	Injury required treatment or resulted in some disability Minor complaints not included	Match only	5.2% or 50.83/1000 players/match	Ankle—58.2% Knee—15.2% Hand—13.3% Other—13.3%	New—71.3% Recurrent—28.7%	Injury incidence higher in highest grades for both Junior (47%) and Senior (35%) players; more hand injuries in junior players; More new injuries occurred in 1st quarter of matches; More recurrent injuries occurred in 2nd quarter of matches; No association between player position and injury; Knee injury resulted in greater disability than other injuries; Most severe injuries were due to slips or falls.
Hopper et al. 1995 [28]	Injury required treatment or resulted in some disability Minor complaints not included	Match only	5.4% or 0.054/player/match	Ankle—84.3% Knee—8.3% Hand—2.8% Other—4.6%	Ligaments—81% Fracture—11% Soft tissue—8%	A grade players higher injury rate than lower grade players; 67% ankle injuries were lateral ligament sprains; 10% ankle injuries were fractures; 65% ankle injuries new (35% recurrent); 80% knee injuries new (20% recurrent); 38% ankle and knee injuries attributed to incorrect landing; Referral to hospital: ankle 15%, knee 27%; More injuries in 1st quarter of matches; injuries greater during defensive play (especially goal defence).
Hopper et al. 1995 [29]	Injury required treatment or resulted in some disability Minor complaints not included	Match only	30.6% of players injured	Ankle—59.1% Knee—18.2% Spinal—18.2% Achilles—4.5%	Ankle injuries mostly lateral ligament sprains Knee injuries included 3 ACLs	Injury rate higher in Grade A1 (54%) vs other grades (19%); no injuries during training; injury risk higher in more athletic players (higher vertical jump and anaerobic fitness; less hypermobile; less endomorphic); higher jump ability and less endomorphy explained difference in injury risk between Grade A1 and other grade players; more severe knee injuries than ankle injuries. Back injuries associated with player contact; ankle and knee injuries associated with landings.
Maulder et al. 2013 [30]	Time loss training or game No history lower limb injury in previous 6 months	Match and training	37.5% of players injured	Ankle—22.2% Calf—22.2% Knee—22.2% Achilles—11.1% Adductor—11.1% Shin—11.2%	Ankle sprain—22% Calf strain—22% Patella tendonosis—22% Achilles strain—11% Adductor strain—11% Shin splints—11%	Asymmetry in turn performance > 10% associated with increased lower limb injury risk.
McKay et al. 1996 [31]	Bodily harm resulting in stopped play, substitution, or obvious disability	Match and training	17.3 injuries/1000players/season Major or severe injuries 1/250 games	Lower limb—65.9% Upper limb—25.7% Trunk—4.7% Head/neck—3.9%	Sprain/strain—62.9% Bruise/laceration—17.6% Other—19.5%	Ankle 30.2%, Knee 17.8%, calf/shin 10.1%, hand 20.9%, back 4.7%. More severe injuries in netball (3.3×) than basketball; Collisions (13.9%), falls (12.6%), poor landings (15.1%), and being hit by the ball (18.2%) were the main injury inciting events.
McManus et al. 2006 [32]	Bodily harm resulting in decrease in sports activity, required medical advice or treatment, or had adverse economic or social consequences; Recurrences counted as original only; No injury in previous 3 months	Match and training	14/1000 PH	Ankle—32% Knee—17% Hand/wrist—15% Back—9%	Sprains—34% Strains -22% Bruising—15%	Risk factors for injury: no warm-up (IRR 1.11) and not being open to new ideas (IRR 1.04). Protective factors regarding injury: training > 4 h/week (IRR 0.66) and having no injury in the previous 12 months (IRR 0.58).
Pickering Rodriguez et al. 2017 [33]	Non-contact bodily harm to soft tissues of lower limb and time loss >1 game	Match and training	34% injured 11.29 lower body injuries/1000 PH Elite 19.35/1000 PH Sub-elite 7.13/1000 PH	Calf—33% Ankle—25% Knee—17%	Included strains, sprains, tears, avulsion fractures, impingement, inflammation	Higher stiffness in soleus and Achilles increased non-contact lower limb injury risk in elite players only.
Pringle et al. 1998 [34]	Bodily harm that impaired a player’s performance	Unclear	13/1000 PH Moderate injuries 6/1000 PH	Ankle Wrist Fingers	Sprains most common 8 minor injuries 7 moderate injuries	Incidence of injury in children playing sport is low.
Stevenson et al. 2000 [21]	Bodily harm resulting in decrease in sports activity, required medical advice or treatment, or had adverse economic or social consequences	Match and training	12.1/1000 PH 20% of players had an injury requiring medical attention	–	37% injuries were ligament strains or tears Moderate-severe—20%	Injured players slightly older than non-injured. Highest rate of injuries occurs in first month of season—IR 28.9/1000 player hours. 20% of netball injuries were graded as moderate-severe but few required hospital admission. 93% of players were female.
Zulkarnain et al. 2019 [35]	Self-reported lower limb injuries during training or competition	77 h training 32 h matches	Overall 5.9/1000 PH Training only IG 5.0/1000 PH Training only CG 9.7/1000 PH	Ankle 65% Knee 35%	Sprain 71% Strain 29%	Intervention reduced training injuries but not match injuries or re-injuries. Number needed to treat to reduce lower limb training injuries was 3.
**Prospective tournaments**						
Hopper and Elliot 1993 [17]	A disability to lower limb or back that caused pain or some degree of dysfunction.	Tournament matches	23% of players sustained lower limb or back injuries	Ankle—36.6% Foot—11.5% Calf/shin—19.2% Knee—17.3% Thigh—1.9% Back—13.5%	Sprain—40.5% ACL—3.8% Strain—1.9% Tendinitis—11.5% Fracture—3.8% Spine—9.6% Joints—11.5% Haematoma—7.7% Medical issues—7.7% Grade 1—71% Grade 2—15.4% Grade 3—13.5%	32.5% of players had an injury at start of tournament—Open 44%; U21 27%; U16 27%. New injuries 63.5%; Recurrent 36.5%. Severity: Grade 1 71% of injuries, Grade 2 15.4%, Grade 3 or fracture 13.5%. Common overuse injuries were shin soreness (38%); retropatellar pain (24%; Older > Younger). No significant differences in perceived landing techniques between injured and uninjured players. No relationships identified between types of injury and podiatric variables. Perceived circumstances of injury: landing 29%), player contact (29%), slip/trip/sudden stop (21%).
Hopper 1997 [20]	A disability to lower limb or back that caused pain or some degree of dysfunction.	Tournament matches	24% of players sustained lower limb or back injuries	Lower limb/back—24%	Any	Somatotype did not influence injury incidence. Mid-field players were more mesomorphic; goal defence players were more ectomorphic; no differences in somatotype between levels of competition.
Hume et al. 2000 [36]	Reporting for injury treatment	Tournament matches 5502 player hours	23.8 injuries/1000 PH 139.4 injuries/1000 players 0.14 injuries per player	Ankle—14% Knee—14% Leg/thigh—19% Foot—5% Upper limb—13% Torso/pelvis—19% Head/face—5%	Sprain—37% ACL rupture—2% Muscle strain—17% Bruise/contusion—18% Graze/abrasion—5% Shin soreness—3% Joint—3% Other—15%	Higher incidence of injuries in younger vs older players; ankle and knee injuries were the most frequent injuries. Injuries were most commonly attributed to incorrect landings, collisions with players, being struck by the ball, or repetitive movements. Behaviours identified that may contribute to injury risk or damage were not warming up, not wearing high-cut netball shoes, and finishing the game before seeking treatment for injury. 35.9% of players reported their injury was recurrent but use of preventative strategies such as wobble boards was poor.
Langeveld et al. 2012 [18]	Any physical complaint that forced the player to receive medical attention. Injury severity based on number of matches missed.	Tournament matches warm-up and practice sessions	500.7/1000 PH 15% of players injured	Ankle—36.1% Knee—18.5% Upper limb—16.1% (fingers 9.3%, wrist 4.9%, hand 1.9%) Lower leg/Achilles—11.7% Other—17.6%	Ligament—46.8% Haematoma—14.8% Muscle—12.3% Meniscal—8.8% Bone (other)—5.4% Minor—> 70%	95% of injuries during matches, 3% during warm-up, 2% during practice sessions; 60.8% of injuries were associated with contact between players. Findings suggest injuries rates higher in tournaments than in regular season games. 27.8% of all injuries were recurrent: 48.7% of ankle, 21.1% of knee, 29% of lower leg/Achilles, 10.5% of finger injuries. Ankle injuries—89% ligament, 38% to lateral ligament. Most injuries minor (> 70%) with player not missing a game; 11.4% missed 1–2 games; 2% missed 3–5 games; more injuries occurred in 2nd and 3rd quarters; goal defence (22%) and centre (17.6%) were positions injured most.
Coetzee et al. 2014 [19]	As per Langeveld et al. 2012	As per Langeveld et al. 2012	500.7/1000 PH 15% of players injured	As per Langeveld et al. 2012	Acute—91.0% Recurrent—8.8%	Factors associated with injury included previous injury, lack of core stability, lack of neuro-muscular and proprioceptive training. Flexibility training uptake high but limited evidence of benefit for injury prevention. Injury incidence higher (1.9×) on cement courts than synthetic surfaces; 80% knee injuries and 89% serious injuries occurred on cement courts.
Smyth et al. 2019 [37]	Any injury requiring physiotherapy attention. Sport incapacity injuries—where player missed part of match or reported reduced capacity.	9 × 40 min matches per team	Injury incidence 89.4/1000 PH U19 > U17 IRR 1.64 Sports incapacity 19.1/1000 PH	Ankle 25% Foot 12.6% Trunk 16.5% Lower leg 9.7% Head & neck 5.8% Wrist & hand 5.8% Pelvic area 4.9% Shoulder 4.9% Thigh 2.9%	Ankle sprains 13.6% Foot blisters 10.7% Lumbar pain 9.7% Incapacity 21% of injuries: Ankle *n* = 4 ACL rupture *n* = 3 Concussion *n* = 3 New > recurrent (IRR 9.3) Trauma 51, Overuse 42 (IRR 1.24)	Main body region injured was lower limb. Joint injuries were 33% of all injuries. Main circumstances of injury were collision (16.5%), contested landings (12.6%), and running (15.5%). 27% of players arrived at tournament with an existing injury or illness.
**Retrospective**						
Attenborough et al. 2016 [38]	Recurrent: two or more sprains to the same ankle		72% previous ankle sprain 47% recurrent ankle sprain	Ankle injuries	Sprains	High prevalence of chronic ankle instability as indicated by recurrent sprains and perceived ankle instability.
Finch et al. 2006 [39]	Injury: any injury associated with participation Significant injury: required treatment, interfered with daily activities and/or adverse effects on participation or performance in subsequent activity	Participation included in survey	19 injuries/10,000 population 51 injuries/1000 participants 24 significant injuries/1000 participants in netball Participation rate for netball 389/10,000 population	All injuries and significant injuries	Any	Conclusions related primarily to comparisons among sports; prevention should be aimed at sports with large participation including netball. Netball had a lower rate of injury than cricket, horse riding and soccer but more than Australian football, basketball and tennis. Children lower overall injury rate but more likely to require treatment.
Hopper and Elliot 1993 [17]	A disability to lower limb or back that caused pain or some degree of dysfunction.		23% of all players sustained lower limb/low back injuries	Right ankle 66.7% Left ankle 49.6% Right knee 25.9% Left knee 17.1% Retropatellar pain 23.7% Shin soreness 38.2% Back pain 33%	Grade 1—71% Grade 2—15.4% Grade 3—13.5%	Ankle (58%), knee (22%) and overuse injuries common in netball players. Back pain more common in Open (11.8%) and U21 (12.2%) than U16 (8.7%) players. Perceived reasons for injuries: ankle—incorrect landing; knee—slip, trip or sudden stop. A number of relationships were identified between types of injuries and podiatric variables. Also, only 23% of players had normal foot types, 43% had rear foot varus, 20% had pronated foot postures, 14% had other foot types.
Hopper et al. 1994 [40]	A disability to lower limb that caused pain or some degree of dysfunction.		90% > lower limb injuries during career	Both ankles 36% One ankle 16% Both knee 6% One knee 13% Shin 18% Retropatellar 11%	No injury—8% 1 injury—23% 2 injuries—32% 3 injuries—22% 4 injuries—9% 5–6 injuries—5%	98% of players had symmetrical foot types; 90% experienced at least one lower limb injury. Bilateral ankle injuries common whereas as unilateral knee injuries more common. Pronating foot types with rear foot abnormalities were the most common foot types and the types most associated with lower limb injuries.
Pillay et al. 2012 [41]	Any physical complaint that occurred during a match or training, irrespective of medical attention or time loss	Matches 2/week Netball training 3.7/week Gym training 3.4/week	1.9 injuries/player/season 61.8% of players injured/season National 84.3% injured State 59.2% injured Club 54.5% injured	Ankle 37.5% Knee 28.6% Other leg 10.6% Upper limb 12.7% Other 6.7%	Mild—40% Moderate—16% Severe—44% 33% miss > 1 game	State (provincial) players sustained more ankle injuries (56.7%) than club (22.5%) or national (20.8%) players. State players had more knee injuries (61%) than national (26%) or club (13%) players. Injuries attributed to landing (ankle 29%, knee 19%) and tripping (ankle 8%, knee 6%). Centre (28%) and goal attack (19%) tended to have higher knee injury rates than other positions.
Singh et al. 2013 [42]	Trauma resulting in cessation of play	62.7% netball training > 6 h/week 91.5% fitness training with 71% > 3 h/week	68% of 59 players injured 29% one injury 27% two injuries 12% three injuries	Ankle 55.8% Knee 41.9% Wrist 2.3% 23.7% had recurrent injuries	Ankle—ligament sprains/Achilles Knee—ACL, meniscus, patella tendon 23.7% recurrent ankle or knee injuries	Ankle—71% lateral ligament sprains; 21% Achilles tendonitis; Knee—22% ACL; 17% meniscal; 33% patella tendon; knee injuries in U21 and Senior players (not U16); 23.7% injuries recurring. Injuries attributed to poor landing (52.5%), collisions (27.5%), playing surface (17.5%), repetitive movements (2.5%). 23.7% of injuries recurring (to ankle or knee). More injuries to wing attack (31.3%) and goal defence (23.5%) positions. 10.2% reported a foot abnormality; 60% wore medium-cut shoes, 37.3% wore low-cut shoes
Smith et al. 2005 [43]	Trauma causing player to cease play and miss > 1 game		35% a netball injury	Ankle 42% Knee 27% Finger 15% Other 16%	Any	Non-hypermobile (*n* = 70) 21% injured; moderately hypermobile (*n* = 51) 37% injured; hypermobile (*n* = 79) 43% injured; players in more hypermobile groups 3× more likely to be injured. Risk of injury increased 1.5× with each year of netball played.
Stuelcken et al. 2016 [44]	Documented ACL injuries		Wing attack 62.5% Centre 19% Goal shooter 12.5% Wing defence 6%	Left knee *n* = 10 Right knee *n* = 6	ACL injuries only	Court position: attacking third 44%; centre third 37%; defensive third 19%. Player: attacking 69%; defending 25%; loose ball 6%. Circumstances: Landing from jump 81% (receiving pass 77%; block/intercept 23%); repositioning 13%, loose ball 6%; 50% non-contact; 50% indirect contact. Most injuries in first or fourth quarters of games.
Whatman et al. 2017 [45]	Overuse injuries defined as those without a specific identifiable event responsible for their onset Substantial problems—moderate to severe reductions in or inability to perform training or competition		Knee problems 31% Substantial knee problems 10% Ankle problems 51% Substantial ankle problems 24%		Overuse knee and ankle problems	Relationships between overuse knee and ankle injuries and movement competency, landing technique, ankle range of motion and jump performance investigated. No relationships identified.
**Hospital, clinic or insurance records**						
Cassell et al. 2003 [46]	Medical treatment of netball injuries in injury surveillance		6.9 (5.4–8.3) % of sport and recreation injuries at ED 6.7 (5.1–8.2) % of sport and recreation injury presentations to General Practitioners		Any	Netball was ranked 4th in emergency department presentations and general practitioner presentations after Australian football, cycling and basketball.
Chong et al. 2004 [47]	Female ACL injuries at hospital		4 of 13 (31%) female ACL injuries were for netball	Knee	ACL only	All reported as non-contact landing injuries; *n* = 3 playing for school (mean age 15 years), *n* = 1 playing for club (25 years); *n* = 3 injured during match, *n* = 1 injured during training.
Fernando et al. 2018 [9]	Netball injury presentations to ED		> 15 years 38.7/100,000 population Females 92% Males 8%			> 4 years *n* = 7777 presentations; 5–14 years 2250 (29%); 15–24 years 3151 (41%), 25–44 years 2139 (28%), > 45 years 193 (2%). Netball ranked 13th out of 20 sports for ED presentations relative to participation.
Finch et al. 1998 [6]	Netball injuries at hospital ED		3.7% child/6.6% adult sports injury ED presentations 3.3% child/2.5 adult sports injury hospital admissions 14% injured children and 8% injured adults admitted to hospital	Children *n* = 1924 Head—5.8% Upper limb—54.4% Lower limb—37.3% Trunk—1.4% Other—1.1% Adults *n* = 3098 Head—4.9% Upper limb—27.5% Lower limb—63.9% Trunk—2.0 Other—1.8%	Lacer/abrasion—3.8% Haematoma—14.9% Inflammation—10.7% Fracture—22.0% Sprain/strain—43.5% Other—4.8% Lacer/abrasion—3.4% Haematoma—9.3% Inflammation—10.0% Fracture—13.3% Sprain/strain—57.5% Other—6.3%	Both child and adult netball injuries mostly sprains, fractures and bruising/inflammation. Upper limb injuries more common in children (54.4%) versus adults (27.5%). Lower limb injuries more common in adults (63.9%) versus child (37.3%). Head injuries similar in children (5.8%) and adults (5%).
Flood et al. 2009 [48]	Netball injuries at hospital		Annual netball hospitalisation rate 1.4 /1000 participants/year Fractures 0.4/1000 participants/year ACL 0.4/1000 participants/year	Knee/leg—37.4% Ankle/foot—21% Upper limb—27.2% Trunk—1.7% Head/neck—6.6% Other—6.2%	Fracture—29.5% Sprain/strain—27.8% Muscle/tendon—17.3% Dislocation—10.1% Other—5.6%	88.9% female. Fractures were most common injury, mostly forearm fractures, and highest in 5–14-year age group; ACL: 98% of admissions for ACL rupture were elective, in private hospitals, in 25–34-year age group. Achilles rupture: rate higher (1.7×) for males; highest in 35–44-year age group.
Gwynne-Jones et al. 2011 [49]	Netball Achilles injury at hospital		Achilles ruptures 24.0/100,000 population	Achilles	Ruptures	Netball: 24% of all Achilles injuries; 31% of sporting Achilles injuries; 54% of Achilles injuries in women 15–40 years.
Hassan et al. 2001 [50]	Fractures occurred participating in sport; resulted in presentation to ED		Netball injuries *n* = 54 Fractures = 24% of hospital netball injuries	Fingers 75%	Fractures *n* = 13	Place of netball injury: school grounds 100%; netball accounted for 12% of fractures in girls, 2^nd^ after rollerblading. Fractures 24% of netball injuries; 75% netball fractures to forearm or fingers. Circumstances of netball injury: 61% struck by ball; 23% fall; 8% collision; 8% other.
Hon et al. 2001 [51]	Sports related fractures at hospital		Netball injuries *n* = 3 2.6% of all sports fractures	Fractures	Fractures *n* = 3	33% of female sports fractures due to netball.
Hume 1993 [23]	Presentations at hospital, ED, or sports injury clinic or Insurance claims		Netball hospitalisation rate 4.3/100,000 population/year 143/100,000 netball players/year 7.7% of sports ED attendances 9.5% netball players injured/year 5.3% sports insurance claims/year 4.5–5.6% netball players injured/year (Clinic data)	**Hospitalisation** Head/Face 8.4% Upper limb 11.2% Lower limb 79% **Emergency Dept** Head/Face 2.5% Upper limb 33.4% Lower limb 49.2% **Insurance claims** Head/Face 19.7% Upper limb 12.3% Lower limb 62.6% **Sports Med Clinic** Head/Face 4.6% Upper limb 24.7% Lower limb 66.2%	**Hospitalisation** Sprain/strain 58.7% Fracture 20.3% Dislocation 11.2% **Emergency Dept** Sprain/strain 63.7% Fracture 9.5% Contusion 7.4% **Insurance claims** Sprain/strain 56.6% Fracture 15.1% Dental 17.0% **Sports Med Clinic** Sprain/strain 73.1% Fracture 7.5% Graze 7.5%	26.2–30% of netball injuries recurrent (Clinic data). Injuries to the ankle, knee and fingers were the most prevalent; injuries to the head/face were more substantial in insurance claims data. Sprains/strains were the most prevalent types of injury; Fractures were more prevalent in hospital/ED and insurance claims data; Dental injuries only captured in insurance claims data. Injury severity of hospitalisations: 53.1% minor, 37.8% moderate, 9.1% severe Hospitalised injuries due to: over-exertion 56.6%; falls 29.3%; struck by person or object 13.3%. Insurance claims due to: tripping/stumbling 22.6%; being struck 17.3%; lifting/straining 15.5%; loss of balance 14.8%. Minor injuries more likely treated as Sports Injury Clinic. Slightly more injuries in wing defence 19.4% and centre 18.7% versus< 15% other positions Occurrences varied during warm-up (3%); 1st half 43.3%; 2nd half 44.8%; practice 1.5% 89.5% of netball hospitalisations to females; 10.5% to males.
Hume et al. 1994 [24]	Presentations at hospital, ED, or sports injury clinic or Insurance claims		89.34/100,000 population	All	Lower extremity most frequent For ACC claims: sprains/strains dominant	Netball 4th most injuries after rugby union, rugby league and soccer.
Joseph et al. 2019 [3]	Injury resulting from trauma while training for or playing netball in approved competition		2.936 claims/1000 participants	Knee 42% Ankle 29% Wrist 11%	Sprains/ligaments 57% Fractures 15%	Most claims in 18–24 years (25%) and 25–34 years (30%) age groups. Slightly more injuries in 2nd quarter of match, but more injuries in quarters 1, 2 and 3 than 4. 92% of injuries occurred during matches.
King et al. 2019 [52]	Any injury as a result of sports participation assessed and reported by a registered health practitioner		11748 moderate-to-serious injuries 9 serious injuries	Head & neck 2.4% Upper limb 10.4% Lower limb 82% Other 0.6%	Soft tissue 81.7% Fracture/dislocation 16.7% Concussion/brain 0.9% Laceration 0.4% Dental 0.14%	Main findings related to costs of injuries.
Kirkwood et al. 2019 [8]	Injury related to playing netball		157 injuries	Lower Limb 36.4% Upper Limb 22.7% Head 2.6%	Fractures 19.5% Ligament damage 2.6% Concussion 0.65%	Only one admission to hospital for a severe injury related to netball.
Love et al. 1998 [53]	New dental claims that year Minor—Payment to health professional for treatment; no payment to claimant		260 netball dental claims/year	Dental	Dental	Netball one of 45 sports and one of top 10 with dental claims. Most dental injury claims were new claims.
Otago et al. 2007 [54]	Insurance netball injury claim accepted		9.49 injuries/1000 players	Lower limb—85.3% Upper limb—8.7% Spine/torso—3.1% Head/face—2.9%	Ankle sprain 31% Knee sprain 20.5% Knee reconstruct 13.6%	Injury costs: Knee 56.9%; ankle 12.7%; calf/Achilles 11.8%. Recommended injury prevention focus on ankle ligament sprains, knee ligament sprains and Achilles tendon strains; focus on Achilles injury should be in players >25 years Injury claim rate highest in 30–39-year group; least in 10–14-year group; claim costs highest in 25–29-year and > 40-year groups; least in 10–14-year group.
Purdam 1987 [16]	Treatment in physiotherapy department		5.25 injuries/player/year	Ankle—13.3% Knee—12.4% Lower limb—43.8% Upper limb—3.8%	Extrinsic—23.9% Intrinsic—26.7% Overuse—30.5% Spinal—20.0%	Author noted the relatively high incidence of calf (10%) and shin (12%) problems in netball players and suggested the training surface may have contributed. Ankle taping/bracing and preventative wobble board program recommended but identified compliance with recommendations an issue.
Smartt et al. 2009 [55]	Public hospital inpatients		5 injuries/100,000 participants	Lower leg/knee 57% Forearm/elbow13% Wrist/hand 8% Ankle/foot 6%	Muscle/tendon 46% Fracture 32% Sprain/strain 7%	81% female (19% male); injury numbers peaked in 30–34-year age group for males and females Also, a peak in female injury numbers in 10–14-year age group; overall injury rate increased with age, with highest injury rate in 35–49-year age group; 0–14 years—forearm fractures dominant; > 14 years—Achilles dominant.

*ACL* anterior cruciate ligament; *AFL* Australian Rules Football; *C* centre; ED emergency department; *GA* goal attack; *GD* goal defence; *GS* goal shooter; *IR* injury rate; *IRR* incidence rate ratio; *Lacer* laceration; *PH* player hours; *SEBT* Star Excursion Balance Test (Munro and Herrington, 2010); *WA* wing attack; *WD* wing defence

^a^Exposure: many studies indicated whether calculations incorporating exposure were match, training or combined but few studies reported the exposure data

^b^Key findings: reporting injury rates was not the primary aim of all studies, so key findings may relate to risk factors, intervention effects, identifying groups with greater or less injury vulnerability

#### Injury Definitions

Only one prospective study [26] did not provide a clear injury definition. Nine (45%) prospective [13, 18, 19, 21, 22, 28, 29, 32, 36] and three (33%) retrospective [38, 39, 44] studies included seeking or receiving treatment in their injury definition. Four (20%) prospective studies [25, 30, 33, 37] and one retrospective study [43] included time loss from training or competition in their definition, with a further three studies using time loss to determine injury severity [18, 19, 45].

#### Player Exposure to Netball Matches or Training

Match and training exposure were reported in eight (57%) of the season competition prospective studies [21, 22, 25, 30–33, 35]. Match only exposure was reported in three (21%) of these studies [13, 28, 29] and all (100%) of the tournament studies [17–20, 36, 37].

#### Injury Rates

A variety of methods were used to report injury rates and incidence. Of the prospective studies, 11 (55%) reported injury incidence per 1000 player hours (PH) [18, 19, 21, 22, 25, 32–37]. Six (30%) studies reported injury rates per player [26, 27, 36], per 1000 players [36], per player/match [13, 28] or per player/season [31]. Four of the season competition prospective studies reported injury rates of 11.3 to 14/1000PH [21, 22] [32, 34] for all injuries. Rates of 1.74/1000PH were reported for ankle sprains [25], 11.29/1000PH for lower limb injuries [33] and 5.9/1000PH for ankle and knee injuries [35]. Greater injury rates for matches (17×) compared with training were reported by Attenborough et al. [25] and in elite (2.7×) compared with sub-elite players [33]. Ten (50%) of the prospective studies [13, 17–21, 28–30, 33] and seven (78%) of the retrospective studies [17, 40–45] reported the percentage (%) of players injured. The % of injured players in regular season play ranged from 5.2–20% in recreational players to 30.6–37.5% in predominantly elite players. Injury proportions in tournaments ranged from 15 to 25%. The hospital/clinic/insurance records studies reported netball injuries as a % of all presenting injuries studied (*n* = 6) [6, 23, 46, 47, 50, 51], relative to population (*n* = 4) [9, 23, 24, 49] or estimated playing population (*n* = 5) [3, 23, 48, 54, 55], or simply stated the number of cases (*n* = 3) [8, 52, 53].

#### Comparison of Injury Rates in Matches, Tournaments, Training and Warm-up

Hopper et al. [29] reported no injuries during training, Attenborough et al. [25] reported nine ankle sprains during matches compared with two during training, and Chong et al. [47] reported three ACL injuries during matches and one during training. Langeveld et al. [18] found that 95% of injuries occurred in matches, 3% in warm-up and 2% in practice and Joseph et al. [3] reported that 92% of injuries occurred in matches. Hume [23] also commented that injuries during warm-up (3%) and practice (1.5%) were low compared with matches. Langeveld et al. [18] noted that injury rates were higher in tournaments compared with regular season competition.

#### Injury Sites

Forty-three studies (96%) provided some data on injury site in terms of body area. There were various methods of categorising body areas among the studies, some more specific (e.g. ankle sprains, Achilles injury), others more generalised (e.g. lower limb injury) (Table 2). Fifteen studies (33% of all 46 studies) were limited to specific body sites or regions based on the aim of their study and eight of those (17% of all studies) were limited to a single structure or injury type, for example, ACL [44, 47], Achilles [49], ankle [25, 38], fracture [50, 51] or dental [53]. Hassan et al. [50] noted that 75% of netball fractures were to the fingers. In those studies that reported the injury site as a percentage of total netball injuries, the range of those percentages were as follows: ankle *n* = 19, range 13.3 to 84.3% [13, 16–18, 22, 26–30, 32, 33, 36, 37, 40–43, 48]; knee *n* = 19, range 8.3 to 50.0% [3, 13, 16–19, 22, 26–30, 32, 36, 37, 40–42]. Injuries to the lower limb *n* = 8, ranged from 36.4 to 85.3% [6, 8, 16, 20, 23, 31, 52, 54], and to the upper limb *n* = 11, ranged from 3.8 to 54.4% [6, 8, 16, 18, 23, 31, 36, 41, 48, 52, 54].

#### Types of Injury

Thirty studies (65.2%) provided indications of the tissue damaged. Seven studies reported data on injury recurrence, with reports of 26.2–36.5% [13, 17, 18, 23, 36] for all injuries, 20% for knee [28] and 35% [28] and 47% [25] for ankle injuries. Hume [23] and Hume and Marshall [24] used multiple categories of injury types ranging from abrasions to dental and intracranial injury types in their analyses of hospital and insurance databases related to netball injuries in New Zealand. Hume [23] noted that head/face injuries are more substantial in insurance claims data than in prospective, retrospective, hospital or clinic data and that dental injuries are primarily only captured in insurance data. Hopper et al. [13] distinguished between new and chronic injuries. Overuse injuries were reported in three studies [16, 17, 45]. Hopper and Elliot [17] found shin soreness (38%) and retropatellar pain (24%) were common. Purdam [16] reported 30.5% of injuries were overuse including 10% of calf and 12% of shin injuries. Whatman et al. [45] found overuse knee and ankle injuries were common in secondary school players.

Ligament sprains (ankle, knee, Achilles) [6, 17, 18, 21–25, 28–38, 42, 54] and to a lesser extent muscle strains (calf, adductor) [6, 17, 18, 22, 23, 30–33, 35, 36, 48, 55] were the most widely reported injury types. Substantial bruises/hematomas/contusions [17, 18, 22, 28, 31, 32, 36] and lacerations/abrasions/grazes [6, 23, 31, 36] were also reported. Smyth et al. [37] reported that 10.7% of tournament players presented with foot blisters. Fractures [6, 17, 18, 22, 23, 28, 33, 48, 50, 51, 55], dislocations [23, 48] and tendon ruptures [17, 37, 42, 44, 49, 54] were far less prevalent but were more evident in the hospital records that focused on more severe injuries. The first study reporting concussions related to netball was published in 2002 [22], and only three of the studies published since have reported on concussions related to netball [8, 37, 52].

#### Severity of Injury

Sixteen studies (34.8%) used descriptive categories, for example, mild, minor, moderate, severe, new, recurrent and overuse. Many authors stated that most netball injuries are minor, but the rates and definitions of minor, moderate and severe injuries varied among studies. Ferreira et al. [27] reported rates of 35% minor, 56.5% moderate and 8.7% severe, and Finch et al. [22] reported 60-70% of injures as moderate severity, indicating that treatment by a health professional was required. Stevenson et al. [21] indicated that 20% of netball injuries were moderate to severe but few required hospitalisation. Hopper and Elliot [17] used a grading system modified from Oakes (1981) [56] and reported 71% of tournament injuries were Grade 1, 15.4% Grade 2, and 13.5% were Grade 3 which included fractures. Langeveld et al. [18] found 70% of injuries were minor (not missing a game), 11.4% resulted in missing 1–2 games, and 2% resulted in missing 3–5 games. Hopper [13] found that knee injuries resulted in greater disability than other injuries, which was supported by Hopper et al. [29] who also found that there were more severe knee injuries than ankle injuries. Hopper et al. [28] reported that 15% of ankle and 27% of knee injuries required referral to hospital and that 10% of ankle injuries were fractures. Whatman et al. [45] reported that 10% and 24% of players had substantial knee and ankle problems, respectively. Otago et al. [54] concluded that injury costs were highest for knee injuries.

McKay et al. [31] found there were more severe injuries in netball than basketball, and Finch et al. [39] reported that children were more likely to require treatment by a health professional than adults. The hospital/clinic/insurance records studies reported primarily on more severe injuries, although Kirkwood et al. [8] noted that there was only one admission to hospital for a severe injury related to netball in their study of 157 injuries. Finch et al. [6] reported fractures were 22% of injuries in children and 13.3% of injuries in adults. Flood et al. [48] found 29.5% of netball hospital presentations were fractures, 27.8% strains/sprains and 10.1% dislocations, which was similar to hospital and emergency department (ED) presentations reported by Hume [23]. Smartt et al. [55] found 32% of netball hospital admissions were due to fractures. Joseph et al. [3] and Kirkwood et al. [8] found 15% and 19.5% of injuries due to fractures, respectively, and King et al. [52] reported 16.7% of injuries were due to fractures or dislocations.

#### Circumstances of Injury

Sixteen studies (34.8%) reported factors associated with the circumstances of injury (COI). Hopper [13] found that most severe injuries were due to slips or falls, which was one of the main circumstances of injury identified by nine studies [13, 17, 18, 20, 23, 29, 31, 41, 50]. The other main circumstances were landings [17, 27–29, 31, 36, 37, 41, 42, 44, 47], collisions/player contact [17, 18, 23, 29, 31, 36, 37, 42, 44, 50], and being hit by the ball [23, 31, 36, 44, 50].

#### Timing of Occurrence of Injuries

Ten (21.7%) studies reported on some aspect of timing of netball injuries. Five studies (10.9%) included data on the time and/or the quarter in the game in which injuries occurred [3, 13, 18, 23, 28]. Hopper et al. [28] reported that most injuries occurred in the 1st quarter of matches, while Hopper [13] reported that more new injuries occurred in the 1^st^ quarter whereas more recurrent injuries occurred in the 2nd quarter. Langeveld et al. [18] reported that most injuries occurred in the 2nd and 3rd quarters with a linear increase from the 1st to 3rd quarters. Stuelcken et al. [44] investigated ACL injuries in 16 players and found that these injuries occurred most frequently in the 1st and 4th quarters (*n* = 6 and 4, respectively). Hume [23] found no difference between injury rates in the first (43.3%) and second (44.8%) halves of matches. Joseph et al. [3] found slightly more injuries in the 2nd quarter but the main trend was for there to be less injuries in the 4th quarter compared with the other three quarters. In reporting injury incidence in four sports, including netball, Stevenson et al. [21] found there was an overall elevated level of injury incidence in the first month of the season with an injury rate of 28.9 injuries per 1000PH compared with the season mean of 12.1 injuries per 1000PH.

#### Environmental and Surface Conditions

Four studies (8.7%) reported on playing surface in relation to injury frequency. Studies reported a lower injury incidence on synthetic surfaces compared with concrete [19], bitumen [13] and grass [13]. Pillay et al. [41] and Joseph et al. [3] stated that the most common surface on which injuries occurred was outdoor concrete, and Coetzee et al. [19] reported this was the surface associated with most (88.9%) serious injuries. Purdam [16] also suggested that the training surface may contribute to injuries.

#### Player Age and Injury Risk

Fifteen studies (32.6%) included data relating injury incidence to age. Most studies reported a higher injury incidence with increasing age [3, 9, 17, 21, 37, 39, 42, 43, 48, 54, 55], one found more injuries in younger players [36] and in others it depended on the specific injuries being investigated [6, 13, 48, 54, 55]. Hopper [13] found more hand and knee injuries in junior players. Finch et al. [6] found more upper limb injuries in children (54.5%) compared with adults (27.5%) but more lower limb injuries in adults (63.9%) compared with children (37.3%). Finch et al. [39] noted that children had a lower overall injury rate compared with adults. Flood et al. [48] found forearm fractures were highest in the 5–14-year age group, ACL ruptures highest in the 25–34-year age group, and Achilles ruptures highest in the 35–44-year age group, and Otago et al. [54] stated that Achilles injuries occur primarily in those over 25 years.

#### Competition Level and Injury Risk

A higher injury incidence was reported in elite or higher grade players compared with lower grade players [13, 28, 29, 41]. Hopper [13] reported that higher grade players had more injuries than lower grade players at both junior and senior levels. Pillay et al. [41] found that state-level players had a higher incidence of ankle and knee injuries than both club level and national level players.

#### Player Position and Injury Risk

Seven studies reported on the specific position or a group of positions (attacking or defending) being played at the time of injury [17, 18, 23, 28, 41, 42, 44]. The player positions more likely to be injured varied with the study. Stuelcken et al. [44] reported that ACL injuries varied with court position with more injuries in the attacking third (44%) and centre third (37%) compared with the defensive third (19%). Hopper and Elliot [17] also reported that more injuries (54%) occurred when playing an attacking position. In contrast, an earlier study [13] reported no association between player position and injury.

#### Other Factors Associated with Increased Injury Risk

A number of factors were reported as increasing risk of injury. Attenborough et al. [25] reported 4.04 times greater odds of sustaining an ankle sprain during netball if a player’s preseason performance on the posterior-medial direction of the Star Excursion Balance Test (SEBT) was ≤ 77.5% of their leg length. Pickering Rodriguez et al. [33] found a relationship between higher soleus and Achilles stiffness (assessed with a validated hop test on a force platform) and higher incidence of lower body injuries in elite netballers. Smith et al. [43] reported significantly increased injury incidence in junior netball players with higher levels of hypermobility. Also, having a lower limb asymmetry in turn performance > 10% was identified as increasing risk of a lower limb injury [30]. Hopper et al. [29] found that more athletic players, as evidenced by higher vertical jump and aerobic fitness, and less endomorphy, were at higher injury risk. Ferreira et al. [27] found that excess body fat, poor balance and agility, and limited explosive power may have contributed to injuries. Chronic ankle instability contributed to increased risk of recurrent ankle sprains in recreational players [38]. A lack of warm-up [32, 36] and a previous injury [19, 32] were also identified as increasing injury risk. Coetzee et al. [19] reported that a lack of core stability and poor utilisation of neuromuscular and proprioceptive training increased injury risk. Hume et al. [36] reported that players failed to adopt recommended injury prevention strategies, such as using wobbleboards, using ankle supports or wearing high-cut netball shoes. Further, 54.7% reported finishing the game before seeking treatment [36]. All of these behaviours may have contributed to higher risk of injury or more severe injury. Smyth et al. [37] reported that 27% of players arrived at their tournament with an existing injury or illness.

#### Factors Not Associated with Increased Injury Risk or Protective

Hopper and colleagues investigated a number of podiatric variables [17, 40], as well as the somatotype [20] and the landing techniques of players [17], and found no relationships with injury risk. Flexibility training was also found to be of limited benefit in preventing injury [19]. Whatman et al. [45] found no relationships between movement competency, landing technique, ankle range of motion or jump performance on overuse knee and ankle injuries. McManus et al. [32] found that not having an injury in the previous 12 months and training more than four hours per week were protective against injury.

### Comparison of Netball Injuries with Other Sports

A number of studies compared netball injury presentations to those of other sports. McKay et al. [31] reported that netball had 3.3 times more severe injuries compared with basketball. Finch et al. [22] found netball had a lower injury rate than Australian Rules Football (AFL), field hockey and basketball. Finch et al. [39] reported less injuries with netball compared with cricket, horse riding and soccer but more than AFL, basketball and tennis. Fernando et al. [9] compared the top 20 sport and recreational activities in terms of injury-related ED presentations; netball ranked 13th in terms of injury rate and 19th in terms of cost per presentation. Kirkwood et al. [8] found netball ranked 2nd to trampoline for sports-related ED attendances in 0-19 year-old females. Cassell et al. [46] found netball ranked 4th in sports-related ED and general practitioner presentations after AFL, cycling and basketball in Australia. Hume et al. [24] also found netball ranked 4^th^ in presentations after rugby union, rugby league and soccer in New Zealand. Hassan et al. [50] found netball was responsible for the largest percentage of fractures presenting to the ED in girls after rollerblading, and Hon et al. [51] found netball accounted for 33% of sports related fractures in females. Gwynne-Jones et al. [49] found netball accounted for 31% of all sports related Achilles injuries and 54% of all Achilles injury ruptures at hospital in females aged 15–40 years. Love et al. [53] found netball was one of the top 10 sports responsible for sports-related dental insurance claims. More recently, King et al. [52] compared the moderate to serious sports-related insurance claims by females across five sports (netball, rugby union, rugby league, football, cricket) and found by far the most claims were for netball but the relative of risk of injury was lowest in netball. Mean cost per claim was 4th in netball, only higher than football for moderate to serious claims but 2nd to rugby union in serious claims.

### Interventions to Reduce Injuries in Netball

Only two studies [26, 35] reported on the outcomes of an injury reduction program. Elphinston et al. [26] used an integrated multidisciplinary sports science and sports medicine approach incorporating screening and evaluation of players followed by individualised technical training and conditioning to achieve a reduction from 1.3 to 0.2 injuries per player. Zulkarnian et al. [35] found that the Down to Earth safe-landing technique program reduced training injuries but not match injuries.

## Discussion

This systematic review presents the first summary of the published literature on netball injuries. Forty-six studies published in 45 papers met the inclusion criteria. Twenty studies were prospective, nine were retrospective, and 17 were analyses of hospital/clinic records or insurance claims. Not surprisingly, all the studies were from British Commonwealth countries with most conducted in Australia, New Zealand or South Africa. Injury rates over a netball season of 11.3–14/1000PH were reported in recreational players whereas in elite players 19.35/1000PH was reported [33]. Injuries were predominantly to the lower limb, particularly the ankle, with ligament sprains the most common injury type. One study reported younger players had more injuries to the upper rather than lower limbs [6]. Severe injuries (those resulting in hospitalisation) were typically fractures, especially in children, and ACL or Achilles ruptures in adult players. There were few head injuries reported in netball. Player characteristics, such as age and level of competition, may influence injury rates. There are few data on male netball players, although the game is still played predominantly by females. Larger prospective studies would be beneficial to further improve our understanding of possible risk factors for injury and the types of injury in netball.

A number of points are clear from the studies included in this review. Firstly, 87% of the studies were conducted in Australia, New Zealand or South Africa, with very little representation from the other 73 countries who have netball organisations. Australia and New Zealand have, in recent times, been the two dominant nations in international netball competitions. This success may reflect the high proportion of these nations’ young and recreational populations participating in netball. High participation incurs an associated injury impact and cost, and therefore, possible available funding from governments to research injury incidence, type and risk factors in netball.

Second, most studies included players across a wide age range with little reporting of the results by age. Children (< 16 years) were included in 92% of the database studies, 63% of the retrospective studies, but only 39% of the prospective studies. It is generally acknowledged that hospital and insurance data do not provide a complete picture of the volume or type of injuries that occur in a sport but do identify those that are the most serious [6]. Finch et al. [6] provide the most comprehensive hospital emergency department data comparing child and adult injury rates in netball. They reported children experiencing a much higher percentage of upper limb injuries (54% vs 27.5%) and fewer lower limb injuries (37.3% vs 63.9%), more fractures (22% vs 13.3%) and fewer sprains/strains (43.5% vs 57.5%) compared with adults. Only Smartt et al. [55] reported their hospital admission results across a number of age ranges. Among those < 18 years, they identified a higher injury rate in the 10–14-year group; however, most injuries occurred in those 25–39 years with the highest number of cases in the 30–34-year age group. Less serious injuries, which are the most numerous, are usually managed in other ways and at non-hospital locations. Pringle et al. [34] focused on children (6–15 years) in their prospective study and concluded that the incidence of injury in children playing netball is low. There is clearly a need to improve our understanding of less severe injury types and rates by age in netball.

Third, competition level was reported to influence injury rates, which has also been described in other sports [57, 58]. Studies of elite players are often overrepresented relative to less competitive players in sports injury studies [59]. Elite or sub-elite players were the focus of 63% of the retrospective studies, 50% of the prospective studies but only one (8%) of the database studies. Four studies [13, 28, 29, 41] reported that injury rates were higher in players in elite compared with lower competition levels. One limitation when comparing injury rates in elite vs recreational players is that injury data collection methods may differ with competition level, even within the same study [25, 33]. No studies have investigated whether injury rates or types vary with competition levels when elite/sub-elite players are excluded.

A number of potential risk factors for injury in netball, in addition to age and competition level, have been investigated, mostly in only one or two studies. Those reporting increased risk of injury included poor balance [31, 44] and agility [44], joint stiffness [40] or hypermobility [45], lower limb asymmetry [33], chronic ankle instability [35], lack of core stability [20], lack of warm-up [39, 43], previous injury [20, 39] and playing when injured [32, 43], and not adopting recommended injury prevention strategies [20, 43]. Whether player position influences risk of injury is not yet clear [16, 18, 19, 24, 29, 37, 54]. Further studies are required to determine the impact of these factors on injury risk and whether interventions targeting these factors can reduce injuries.

The method of injury data collection used in the studies varied, with many studies using unqualified personnel, for example students or team managers, and this reporting was particularly influenced by the level of competition of the players. In the season-competition prospective studies, those conducted on recreational players only obtained their data by self-report [21, 22, 32]. In contrast, in studies of elite players, almost all injuries were assessed and recorded by physiotherapists [17, 20, 26–29]. In studies investigating injuries across a broad range of competition levels, some were recorded by physiotherapists [13, 28, 29] while others were self-reported or recorded by physiotherapy or sports science students or first aid staff [18, 19, 25, 31, 36]. In the tournament prospective studies, medical staff contributed to injury recording in two studies [17, 20] whereas a range of personnel provided the treatment or completed the injury records in the other studies [18, 19, 36]. Thus, there is uncertainty in the accuracy of injury records obtained from some of these varied methods. The method of data collection most likely to capture all injuries that occur on courts in competition (at all levels) is to have data collected on the courts at the time of play by appropriately qualified sports medicine professionals, with follow-up and specialist referral when necessary. To increase the accuracy and depth of injury data collected, future studies should ensure that all personnel collecting data are suitably qualified and trained in standardised injury reporting, and/or that each injury is reviewed by a qualified health professional.

Injury rates were reported using a number of different metrics and some studies focused on particular types of injury only; consequently, consolidating the findings on injury rates is limited. Some of that inconsistency may be related to the fact that 13 of the articles included in this review were published before 2000 [6, 13, 16, 17, 20, 23, 24, 28, 29, 31, 34, 40, 53], and two before 1990 [13, 16]. Reporting injuries by exposure (/1000PH) was limited in the studies included in this review but data from four prospective studies [21, 22, 32, 34] in recreational players provided consistent values of 11.3 to 14 injuries/1000PH. Injury rates in tournaments or in more elite players appear to be higher. Pickering Rodriguez et al. [33] reported injury rates of 19.35/1000PH for elite players compared with only 7.13/1000PH for sub-elite players. Higher injury risk at higher levels of competition appears to be consistent with several other sports (e.g. tennis, soccer, and rugby union 15 s) [60–62].

Future studies should aim to use standardised methods, such as those outlined by Fuller et al. [63] in their consensus statement for the collection of injury data in rugby union, to further build on the existing evidence provided by the studies in this review. This consensus statement provides operational definitions for injury, severity, injury recurrence, match and training exposure, as well as recommended methods for collecting and classifying injury data. The development of a consensus statement by the International Netball Federation would assist in this process.

Although the proportion of injuries to each body region varied among studies, most netball injuries in those over 15 years were to the lower limb, particularly the ankle and knee. Ankle injuries were far more frequent than knee injuries with the exception of Maulder et al. [30]. There were reports of injuries to other parts of the lower limb [17, 18, 30, 33, 36, 40, 41] and back [17, 20, 22, 26]; the upper limb, particularly the hand [13, 18, 28, 32, 34, 43, 50, 55], wrist [18, 32, 34, 42, 55] and forearm [55]; and relatively smaller numbers of injuries to the head, face or neck [22, 23, 26, 27, 31, 36, 48, 53, 54] although those to the head or face, including dental injuries, were evident in insurance claims [23, 53]. Interestingly, while only four studies included in this review [8, 22, 37, 52] provided any data on concussion injuries in netball, a report on sports-related concussion identified that the rate of concussion in netball has been increasing [64]. Netball Australia has developed a concussion policy [65] based on the Concussion in Sport Australia Position Statement [66] and the Consensus Statement of the International Conference on Concussion in Sport [67]. This concussion policy may be justified as ‘collision’ is a frequently cited circumstance of injury in netball, and females may be more vulnerable to and take longer to recover from a concussion [66].

It was not possible to determine the extent to which there are multiple injuries associated with a single injury incident. For example, it is possible that slips, trips, falls or collisions could result in injuries to both upper and lower limbs and possibly the head. It is not clear in the prospective and retrospective studies whether all injuries are recorded or only the most severe or memorable injuries. Similarly, in hospital records and insurance claims, it is likely only the most severe injury is recorded.

There has been limited standardisation of reporting of COI. In the Coetzee et al. [19] study, 60.8% of injuries were attributed to ‘physical contact’ and Hon and Kock [51] also used this terminology, as did Pringle et al. [34] and Hopper et al. [29]. ‘Collision’, a similar term, was used by Singh et al. [42] and McKay et al. [31]. Landing ‘incorrectly’ and ‘awkwardly’ were used by a number of authors but without a clear definition of a ‘correct’ landing. A more specific system of describing COI may lead to further associations with specific injuries and, as a result, enable equipment, coaching and training strategies to minimise these injuries. This should include intrinsic and extrinsic factors as well as differentiating between acute and overuse injuries [16]. It should also be noted that many studies used questionnaires to gather this information from players, which introduces recall bias and player opinion about what their COI might have been. Players may need training in the terms to use to describe these circumstances to improve consistency of description. Stuelcken et al. [44] was the only study to review video footage of games, providing the opportunity to assess possible mechanisms of ACL rupture, in addition to COI. Injury risk is likely a multi-factorial phenomenon; however, if the circumstances associated with injuries are reported in a detailed, standardised manner, then further studies can examine these more closely. These findings could provide the basis for strategies to be developed for modifiable factors, such as neuromuscular control in ACL injuries in a range of sports [68] and implemented in training for the purpose of reducing the incidence of those injuries.

Currently, most recreational netball competitions are played outdoors on concrete/asphalt ‘all weather’ courts, and so players are exposed to a variety of weather conditions that may influence the type and incidence of injuries [3]. Elite competitions are also often played on these courts, as well as indoors on sprung timber courts, which may produce different injury profiles. Indoor competitions mostly play on synthetic surfaces over concrete bases, again possibly affecting the pattern of injury and frequency. Playing surface has been shown to influence injury rates in netball [3] and other sports [69–71]. Although concrete playing surfaces may be a risk factor for injury in netball [3, 16], data on playing surfaces were limited in the studies in this review and so the impact of playing surface type on injury incidence needs further investigation.

Training data should be reported in future studies to clarify which practices associated with training are associated with injury risk. This should include the type, duration, intensity and frequency of training activities, as well as any injuries that may occur during training. These studies may then be able to identify training practices associated with high injury risk, or the effect of changed training loads on the incidence of injury during games, enabling coaches and trainers to modify training practices and reduce injury risk.

The main strengths of this review are the comprehensiveness of the search, the good agreement between reviewers for inclusion of studies, the stratification of the studies into categories and the rigour of data extraction. The main limitations are those that relate to the quality and extent of reporting in the included studies, particularly differences in injury definitions, limited or lack of clarity regarding the use of health professionals to collect data or verify injury diagnosis in recreational players, reliance on self-report data in many studies, and limited reporting of injuries by age or sex.

Recommendations for research and practice are 1) for data on recreational players to incorporate a diagnosis by a qualified health professional; 2) for netball injury data to be collected by people trained in injury reporting and ideally stored in a centralised national database; 3) to report data by age group, especially among those < 18 years of age, such as (a) < 10 years, (b) ≥ 10 ≤ 15 years and (c) > 15 ≤ 17 years; 4) to provide data on male players separately if included; 5) to provide more detail on the types of head injuries sustained by netball players, including the extent and severity of concussion injuries; 6) to provide greater documentation of injuries relative to training and/or match load; and 7) to provide stronger evidence of risk factors, particularly in relation to circumstances and mechanisms for netball injuries that can be targeted by prevention strategies and provide teachable suggestions for coaches. This would improve the data available to netball clubs, organisations and sports medicine personnel working with netball communities, and enable them to provide appropriate resources and equipment to manage injuries and reduce injury risk in this large, varied population of netball players.

## Conclusions

This review found that most netball injuries are to the lower limb, particularly the ankle, and that injuries to the upper limb have a higher rate in those < 16 years old. Severe injuries are fractures in younger players and ACL and Achilles injuries in adults. Injury rates appear higher in elite players than recreational players. Factors often associated with injury include circumstances such as landings, slips/trips/falls, collisions/player contact and being hit by the ball. Further studies should be directed towards recreational netball, reporting on injury incidence in the under 15-year population and utilising high-quality, standardised methods and criteria. Specific injury diagnosis and a better understanding of the link with circumstances of injury would provide more meaningful data for developing prevention strategies. Further classification of data into acute/recurrent/chronic and timing of injuries may provide links to elements in training and games which increase or decrease injury risk. Studies identifying successful injury prevention strategies are also needed.

## Supplementary Information

**Additional file 1.**

## Data Availability

All data generated or analysed during this study are included in this published article and its supplementary information files.

## Abbreviations

ACL: Anterior cruciate ligament
AFL: Australian Football League
AUS: Australia
C: Centre
COI: Circumstances of injury
ED: Emergency department
GA: Goal attack
GD: Goal defence
GS: Goal shooter
GP: General practitioner
INF: International Netball Federation
IR: Injury rate
IRR: Incident rate ratio
NIH: National Institutes of Health
NSIC: National Sports Information Centre
NZ: New Zealand
PH: Player hours
SA: South Africa
SEBT: Star Excursion Balance Test
TRIPP: Translating Research into Injury Practice Prevention
U: Under
UK: United Kingdom
USSA: University Sports South Africa
WA: Wing attack
WD: Wing defence

## References

[1] International Netball Federation. International Netball Federation Website [https://netball.sport/]. 2019 [updated 01/10/2019.

[2] All Australia Netball Association (2015). All Australia Netball Association.

[3] Joseph C, Naughton G, Antcliff A (2019). Australian netball injuries in 2016: An overview of insurance data. J Sci Med Sport..

[4] Egger G. Sport injury in Australia: Causes, cost and prevention. A report to the National Better Health Program. 1990.

[5] Fong DT, Hong Y, Chan LK, Yung PS, Chan KM (2007). A systematic review on ankle injury and ankle sprain in sports. Sports Medicine..

[6] Finch C, Valuri G, Ozanne-Smith J (1998). Sport and active recreation injuries in Australia: evidence from emergency department presentations. British Journal of Sports Medicine..

[7] Everison R, Leeds M, Wicks S (2010). Sports injuries to children. Kidsafe WA.

[8] Kirkwood G, Hughes TC, Pollock AM (2019). Results on sports-related injuries in children from NHS emergency care dataset Oxfordshire pilot: an ecological study. Journal of the Royal Society of Medicine..

[9] Fernando DT, Berecki-Gisolf J, Finch CF (2018). Sports injuries in Victoria, 2012-13 to 2014-15: evidence from emergency department records. Med J Aust..

[10] Netball Australia. [Available from: www.netball.com.au.].

[11] Finch C (2006). A new framework for research leading to sports injury reduction. Journal of Science and Medicine in Sport..

[12] Moher D, Liberati A, Tetzlaff J, Altman DG, Group P (2009). Preferred reporting items for systematic reviews and meta-analyses: the PRISMA statement. PLoS medicine..

[13] Hopper D (1986). A survey of netball injuries and conditions related to these injuries. Australian Journal of Physiotherapy..

[14] McHugh ML (2012). Interrater reliability: The kappa statistic. Biochemia Medica (Zagreb)..

[15] National Heart LaBI. Study Quality Assessment Tools [Available from: www.nhlbi.nih.gov/health-topics/study-quality-assessment-tools.

[16] Purdam C (1987). A survey of netball and basketball injuries. Excel..

[17] Hopper D, Elliott B (1993). Lower limb and back injury patterns of elite netball players. Sports Medicine..

[18] Langeveld E, Coetzee FF, Holtzhausen LJ (2012). Epidemiology of injuries in elite South African netball players. South African Journal for Research in Sport, Physical Education & Recreation.

[19] Coetzee D, Langeveld E, Holtzhausen L (2014). Training habits, training surface and injuries among South African netball players. South African Journal for Research in Sport, Physical Education & Recreation.

[20] Hopper DM (1997). Somatotype in high performance female netball players may influence player position and the incidence of lower limb and back injuries. British Journal of Sports Medicine..

[21] Stevenson MR, Hamer P, Finch CF, Elliot B, Kresnow M (2000). Sport, age, and sex specific incidence of sports injuries in Western Australia. British Journal of Sports Medicine..

[22] Finch C, Da Costa A, Stevenson M, Hamer P, Elliott B (2002). Sports injury experiences from the Western Australian Sports Injury Cohort Study. Aust N Z J Public Health..

[23] Hume PA (1993). Netball injuries in New Zealand. New Zealand Journal of Sports Medicine..

[24] Hume PA, Marshall SW (1994). Sports injuries in New Zealand: exploratory analyses. New Zealand Journal of Sports Medicine..

[25] Attenborough AS, Sinclair PJ, Sharp T, Greene A, Stuelcken M, Smith RM (2017). The identification of risk factors for ankle sprains sustained during netball participation. Physical Therapy in Sport..

[26] Elphinston J, Hardman SL (2006). Effect of an integrated functional stability program on injury rates in an international netball squad. Journal of Science & Medicine in Sport..

[27] Ferreira MA, Spamer EJ (2010). Biomechanical, anthropometrical and physical profile of elite university netball players and the relationship to musculoskeletal injuries. South African Journal for Research in Sport, Physical Education & Recreation.

[28] Hopper D, Elliott B, Lalor J (1995). A descriptive epidemiology of netball injuries during competition: a five year study. British Journal of Sports Medicine..

[29] Hopper DM, Hopper JL, Elliott BC (1995). Do selected kinanthropometric and performance variables predict injuries in female netball players?. J Sports Sci..

[30] Maulder PS (2013). Dominant limb asymmetry associated with prospective injury occurence. South African Journal for Research in Sport, Physical Education & Recreation..

[31] McKay GD, Payne WR, Goldie PA, Oakes BW, Stanley JJ (1996). A comparison of the injuries sustained by female basketball and netball players. Australian Journal of Science & Medicine in Sport..

[32] McManus A, Stevenson MR, Finch CF (2006). Incidence and risk factors for injury in non-elite netball. Journal of Science & Medicine in Sport..

[33] Pickering Rodriguez EC, Watsford ML, Bower RG, Murphy AJ (2017). The relationship between lower body stiffness and injury incidence in female netballers. Sports Biomechanics..

[34] Pringle RG, McNair P, Stanley S (1998). Incidence of sporting injury in New Zealand youths aged 6-15 years. British Journal of Sports Medicine..

[35] Jaafar Z, Khairullina K. The pilot study on Down to Earth (D2E) injury prevention program among varsity netball players. Gazzetta Medica Italiana Archivio per le Scienze Mediche. 2019;178.

[36] Hume PA, Steele JR (2000). A preliminary investigation of injury prevention strategies in Netball: are players heeding the advice?. Journal of Science & Medicine in Sport..

[37] Smyth EA, Piromalli L, Antcliff A, Newman P, Waddington G, Weissensteiner JR, et al. A prospective study of health problems at the 2018 17/U and 19/U Australian National Netball Championships with comparison of surveillance methodology. J Sci Med Sport. 2019. 10.1016/j.jsams.2019.10.004 31704027

[38] Attenborough AS, Sinclair PJ, Sharp T, Greene A, Stuelcken M, Smith RM (2016). A snapshot of chronic ankle instability in a cohort of netball players. Journal of Science & Medicine in Sport..

[39] Finch C, Cassell E (2006). The public health impact of injury during sport and active recreation. Journal of Science & Medicine in Sport..

[40] Hopper D, Bryant A, Elliott B (1994). Foot types and lower limb injuries in elite netball players. J Am Podiatr Med Assoc..

[41] Pillay T, Frantz JM (2012). Injury prevalence of netball players in South Africa: the need for injury prevention. South African Journal of Physiotherapy..

[42] Singh P, Mansingh A, Palmer W, Williams EW (2013). Injuries in elite Jamaican netballers. West Indian Medical Journal..

[43] Smith R, Damodaran AK, Swaminathan S, Campbell R, Barnsley L (2005). Hypermobility and sports injuries in junior netball players. British Journal of Sports Medicine..

[44] Stuelcken MC, Mellifont DB, Gorman AD, Sayers MG (2016). Mechanisms of anterior cruciate ligament injuries in elite women's netball: a systematic video analysis. J Sports Sci..

[45] Whatman C, Reid D (2017). Movement quality, physical performance and prevalence of overuse injuries in secondary school netball players. New Zealand J Sports Med.

[46] Cassell EPF, C.F.; Stathakis,V.Z. Epidemiology of medically treated sport and active recreation injuries in the Latrobe Valley, Victoria, Australia. Br J Sports Med. 2003;37:405-409. 10.1136/bjsm.37.5.405 PMC1751356 14514530

[47] Chong RW, Tan JL (2004). Rising trend of anterior cruciate ligament injuries in females in a regional hospital. Annals of the Academy of Medicine, Singapore..

[48] Flood L, Harrison JE (2009). Epidemiology of basketball and netball injuries that resulted in hospital admission in Australia, 2000-2004. Medical Journal of Australia..

[49] Gwynne-Jones DP, Sims M, Handcock D (2011). Epidemiology and outcomes of acute Achilles tendon rupture with operative or nonoperative treatment using an identical functional bracing protocol. Foot & Ankle International..

[50] Hassan I, Dorani BJ (2001). Sports related fractures in children in north east England. Emergency Medicine Journal..

[51] Hon WH, Kock SH (2001). Sports related fractures: A review of 113 cases. J Orthop Surg.

[52] King D, Hume PA, Hardaker N, Cummins C, Gissane C, Clark T (2019). Sports-related injuries in New Zealand: National Insurance (Accident Compensation Corporation) claims for five sporting codes from 2012 to 2016. British Journal of Sports Medicine..

[53] Love RM, Carman N, Carmichael S, MacFadyen E (1998). Sport-related dental injury claims to the New Zealand Accident Rehabilitation & Compensation Insurance Corporation, 1993-1996: analysis of the 10 most common sports, excluding rugby union. N Z Dent J..

[54] Otago L, Peake J (2007). The role of insurance data in setting priorities for netball injury prevention strategies. Journal of Science & Medicine in Sport..

[55] Smartt P, Chalmers D (2009). Obstructing the goal? Hospitalisation for netball injury in New Zealand 2000-2005. New Zealand Medical Journal..

[56] Oakes BW (1981). Acute soft tissue injuries: nature and management. Aust Fam Physician..

[57] Clifton DR, Koldenhoven RM, Hertel J, Onate JA, Dompier TP, Kerr ZY (2017). Epidemiological patterns of ankle sprains in youth, high school and collegiate football. Journal of Sports Medicine..

[58] Barber Foss KD, Le Cara E, McCambridge T, Hinton R, Kushner A, Myer GD (2017). Epidemiology of injury in men's lacrosse: injury prevention implications for competition level, type of play, and player position. Physician & Sports Medicine..

[59] Yeomans C, Kenny IC, Cahaln R, Warrington GD, Harrison AJ, Hayes K (2018). The Incidence of injury in amateur male rugby union: A systematic review and meta-analysis. Sports Medicine..

[60] Pluim BM, Staal JB, Windler GE, Jayanthi N (2006). Tennis injuries: occurrence, aetiology, and prevention. British Journal of Sports Medicine..

[61] Klein C, Henke T, Platen P (2018). Injuries in football (soccer)—a systematic review of epidemiology and aetiological aspects. German Journal of Exercise and Sport Research..

[62] King D, Hume P, Cummins C, Pearce A, Clark T, Foskett A (2019). Match and training injuries in women’s rugby union: A systematic review of published studies. Sports Medicine..

[63] Fuller CWMGM, Bagate C, Bahr R, Brooks JHM, Donson H, Kemp SPT, McCrory P, McIntosh AS, Meeuwisse WH, Quarrie KL, Raftery M, Wiley P (2007). Consensus statement on injury definitions and data collection procedures for studies of injuries in rugby union. Br J Sports Med.

[64] Finch CF, Clapperton AJ, McCrory P (2013). Increasing incidence of hospitalisation for sport-related concussion in Victoria, Australia. Medical journal of Australia..

[65] Armstrong S (2018). Netball Australia Concussion Policy.

[66] Elkington L, Manzanero S, Hughes D (2019). Concussion in Sport Australia Position Statement.

[67] McCrory P, Meeuwisse W, Dvorak J, Mark Aubry M, Bailes J, Broglio S (2017). Consensus statement on concussion in sport-the 5(th) international conference on concussion in sport held in Berlin, October 2016. British Journal of Sports Medicine..

[68] Hewett TE, Ford KR, Hoogenboom BJ, Myer GD (2010). Understanding and preventing acl injuries: current biomechanical and epidemiologic considerations - update 2010. North American Journal of Sports Physical Therapy.

[69] Lanzetti RM, Lupariello D, Venditto T, Rota P, Guzzini M, Vadalà A (2017). The influence of playing surface on injury risk in Italian elite rugby players. Muscles, Ligaments and Tendons Journal..

[70] Pasanen K, Parkkari J, Rossi L, Kannus P (2008). Artificial playing surface increases the injury risk in pivoting indoor sports: a prospective one-season follow-up study in Finnish female floorball. British Journal of Sports Medicine..

[71] Mears AC, Osei-Owusu P, Harland AR, Owen A, Roberts JR (2018). Perceived links between playing surfaces and injury: A worldwide study of elite association football players. Sports Medicine - Open.

