# Supplementary material for: Injuries in Netball-A Systematic Review
Source: Sports Med Open. 2021 Jan 6;7:3. doi: 10.1186/s40798-020-00290-7 (PMC7788138; doi:10.1186/s40798-020-00290-7)
Supplement: Supplementary file 1 — Additional file 1. [file 40798_2020_290_MOESM1_ESM.docx]

Supplemental Table 1 - Quality Assessment Results per Study

| **Study** | **Research question or objective clearly stated** | **Study population clearly defined and specified** | **Participation rate of eligible persons ≥50%** | **Subjects selected from same/similar population** | **Sample size justification** | **Exposure(s) of interest measured prior to outcome(s)** | **Timeframe sufficient** | **Different level of exposures as related to outcomes are examined** | **Exposure measures clearly defined, valid, and reliable** | **Exposure(s) measured more than once** | **Outcome measures clearly defined, valid, and reliable** | **Outcome assessors blinded to exposure status** | **Loss to follow-up ≤20%** | **Adjusted for potential confounding variables** |
| --- | --- | --- | --- | --- | --- | --- | --- | --- | --- | --- | --- | --- | --- | --- |
| **Prospective**  **Season competition** |  |  |  |  |  |  |  |  |  |  |  |  |  |  |
| Attenborough et al. 2017 (50) | ✓ | ✓ | ✓ | ✓ | ✓ | ✓ | ✓ | ✓ | ✓ | ? | X | ? | ✓ | X |
| Elphinston et al. 2006 (37) | ✓ | ✓ | ✓ | ✓ | X | ✓ | X | X | X | ? | X | X | X | X |
| Ferreira et al. 2010 (38) | ✓ | ✓ | ✓ | ✓ | ✓ | ✓ | ✓ | X | X | ✓ | ✓ | ? | ✓ | X |
| Finch et al. 2002 (22) | ✓ | ✓ | ✓ | ✓ | ✓ | ✓ | ✓ | X | ✓ | ✓ | ✓ | X | ✓ | X |
| Hopper 1986 (13) | ✓ | ✓ | ✓ | ✓ | X | ✓ | ✓ | X | X | ? | ✓ | ? | n/a | X |
| Hopper et al. 1995 (26) | ✓ | ✓ | ✓ | X | X | ✓ | ✓ | X | X | ? | ✓ | X | n/a | X |
| Hopper et al. 1995 (27) | ✓ | ✓ | X | X | X | ✓ | ✓ | X | X | ? | ✓ | ✓ | n/a | X |
| Maulder et al. 2013 (40) | ✓ | ✓ | X | X | X | ✓ | ✓ | X | X | ? | X | ✓ | n/a | X |
| McKay et al. 1996 (51) | ✓ | ✓ | X | X | ✓ | ✓ | ✓ | ✓ | X | ? | X | ✓ | ✓ | ✓ |
| McManus et al. 2006 (45) | ✓ | ✓ | X | X | X | ✓ | ✓ | ✓ | ✓ | ? | X | X | X | X |
| Pickering Rodriguez et al. 2017 (41) | ✓ | ✓ | X | X | X | ✓ | ✓ | ✓ | X | ? | X | ✓ | ✓ | ✓ |
| Pringle et al. 1998 (25) | ✓ | ✓ | X | X | X | X | X | ✓ | X | ? | X | X | ✓ | X |
| Stevenson et al. 2000 (21) | ✓ | ✓ | X | X | ✓ | ✓ | ✓ | ✓ | ✓ | ? | X | ? | X | X |
| Zulkarnain et al. 2019 (55) | ✓ | ✓ | ✓ | ✓ | ✓ | ✓ | ✓ | n/a | X | ✓ | ? | ✓ | ✓ | ✓ |
| **Prospective**  **Tournaments** |  |  |  |  |  |  |  |  |  |  |  |  |  |  |
| Hopper and Elliot 1993 (17) | ✓ | ✓ | ✓ | X | ✓ | ✓ | ✓ | X | X | ? | ✓ | ? | n/a | X |
| Hopper 1997 (20) | ✓ | ✓ | ✓ | X | X | ✓ | ✓ | X | X | ? | ✓ | ✓ | n/a | X |
| Hume et al. 2000 (28) | ✓ | ✓ | ✓ | ✓ | ✓ | ✓ | ✓ | X | X | ? | X | X | n/a | X |
| Langeveld et al. 2012 (18) | ✓ | ✓ | ✓ | X | X | ✓ | ✓ | ✓ | X | ? | X | ✓ | n/a | X |
| Coetzee et al. 2014 (19) | ✓ | ✓ | ✓ | ✓ | ✓ | ✓ | ✓ | ✓ | ✓ | ? | X | X | n/a | X |
| Smyth et al. 2019 (44) | ✓ | ✓ | ✓ | ✓ | ✓ | ✓ | ✓ | n/a | ✓ | ✓ | ✓ | ✓ | ✓ | X |
| **Retrospective** |  |  |  |  |  |  |  |  |  |  |  |  |  |  |
| Attenborough et al. 2016 (52) | ✓ | ✓ | ✓ | ✓ | ✓ | X | X | ? | X | ? | X | ? | n/a | X |
| Finch et al. 2006 (46) | ✓ | ✓ | ? | ✓ | ✓ | ✓ | ✓ | X | X | ? | X | ? | n/a | X |
| Hopper and Elliot 1993 (17) | ✓ | ✓ | ✓ | X | ✓ | ✓ | ✓ | X | X | ? | ✓ | ? | n/a | X |
| Hopper et al. 1994 (42) | ✓ | ✓ | ✓ | X | ✓ | ✓ | ✓ | X | X | ? | X | ? | n/a | X |
| Pillay et al. 2012 (43) | ✓ | ✓ | ✓ | ✓ | X | X | X | X | X | ? | X | ? | n/a | X |
| Singh et al. 2013 (33) | ✓ | X | X | X | X | ✓ | ✓ | X | X | ? | X | ? | ✓ | X |
| Smith et al. 2005 (29) | ✓ | ✓ | ✓ | X | X | ✓ | ✓ | X | X | ? | ✓ | ✓ | n/a | ✓ |
| Stuelcken et al. 2016 (39) | ✓ | ✓ | X | X | ✓ | X | X | X | X | ? | ✓ | ✓ | n/a | X |
| Whatman et al. 2017 (30) | ✓ | ✓ | ✓ | ✓ | ✓ | ✓ | ✓ | n/a | ✓ | ✓ | ✓ | ✓ | ✓ | X |
| **Hospital, clinic or insurance records** |  |  |  |  |  |  |  |  |  |  |  |  |  |  |
| Cassell et al. 2003 (47) | ✓ | ✓ | ✓ | ✓ | ✓ | X | ✓ | X | X | X | ✓ | ✓ | n/a | X |
| Chong et al. 2004 (48) | ✓ | ✓ | ✓ | ✓ | ✓ | ✓ | ✓ | X | X | ? | ✓ | ? | ✓ | X |
| Fernando et al. 2018 (9) | ✓ | ✓ | ✓ | ✓ | ✓ | ✓ | ✓ | ✓ | ✓ | ? | X | X | n/a | X |
| Finch et al. 1998 (6) | ✓ | ✓ | ✓ | ✓ | X | ✓ | X | X | X | ? | X | X | X | X |
| Flood et al. 2009 (32) | ✓ | ✓ | ✓ | ✓ | ✓ | ✓ | ✓ | n/a | X | ? | ? | n/a | n/a | X |
| Gwynne-Jones et al. 2011 (53) | ✓ | ✓ | ✓ | ✓ | ✓ | ✓ | ✓ | X | X | ✓ | ✓ | ? | ✓ | X |
| Hassan et al. 2001 (31) | ✓ | ✓ | ? | ✓ | ✓ | ✓ | ✓ | X | X | ? | X | ? | n/a | X |
| Hon et al. 2001 (49) | ✓ | ✓ | ✓ | ✓ | ✓ | ✓ | ✓ | X | ✓ | ✓ | ✓ | X | ✓ | X |
| Hume 1993 (23) | ✓ | ✓ | ✓ | ✓ | ✓ | ✓ | ✓ | X | X | ? | ✓ | ? | n/a | X |
| Hume et al. 1994 (24) | ✓ | ✓ | ✓ | ✓ | ✓ | ✓ | ✓ | X | X | ? | ✓ | ? | n/a | X |
| Joseph et al. 2019 (3) | ✓ | ✓ | ✓ | ✓ | X | ✓ | ✓ | X | X | ? | ✓ | ? | ✓ | X |
| King et al. 2019 (36) | ✓ | ✓ | ✓ | ✓ | ✓ | ✓ | ✓ | X | X | ? | ✓ | ? | n/a | X |
| Kirkwood et al. 2019 (8) | ✓ | ✓ | ✓ | X | X | ✓ | ✓ | X | X | ? | ✓ | ? | n/a | X |
| Love et al. 1998 (54) | ✓ | ✓ | ✓ | X | ✓ | ✓ | ✓ | X | X | ? | ✓ | ? | n/a | X |
| Otago et al. 2007 (34) | ✓ | ✓ | ✓ | ✓ | X | ✓ | ✓ | X | X | ? | ✓ | ? | n/a | X |
| Purdam 1987 (16) | ✓ | ✓ | ✓ | X | ✓ | ✓ | ✓ | X | X | ? | X | ? | n/a | X |
| Smartt et al. 2009 (35) | ✓ | ✓ | ✓ | X | X | ✓ | ✓ | X | X | ? | ✓ | X | n/a | X |

**Declarations**

**Ethics approval and consent to participate**

Statements regarding ethics approval and consent to participate were included in all the papers in this review.

**Consent for publication**

Not applicable

**Availability of data and material**

All data generated or analysed during this study are included in this published article and its supplementary information files.

**Competing interests**

Christopher Downs, Suzanne J. Snodgrass, Ishanka Weerasekara, Sarah R. Valkenborghs and Robin Callister declare that they have no conflicts of interest relevant to the content of this review.

**Funding**

No funding supported the development of this manuscript.

**Authors’ contributions**

CD – conducted the search, screened articles and extracted data, and drafted the manuscript.

SS – designed the search, screening and data extraction processes, and edited the manuscript.

IW - screened articles, contributed to the quality of assessment of studies and developed figures, and provided feedback on the manuscript.

SRV – updated the search and screening of articles, provided thorough review of the manuscript and final preparation of the figures.

RC - designed the search, screening and data extraction processes, extracted data and development of the tables, revised and edited the manuscript.

**Acknowledgements**

Not applicable

**Authors information**

Not applicable
